# SPT6-driven error-free DNA repair safeguards genomic stability of glioblastoma cancer stem-like cells

**DOI:** 10.1038/s41467-020-18549-8

**Published:** 2020-09-18

**Authors:** Elisabeth Anne Adanma Obara, Diana Aguilar-Morante, Rikke Darling Rasmussen, Alex Frias, Kristoffer Vitting-Serup, Yi Chieh Lim, Kirstine Juul Elbæk, Henriette Pedersen, Lina Vardouli, Kamilla Ellermann Jensen, Jane Skjoth-Rasmussen, Jannick Brennum, Lucie Tuckova, Robert Strauss, Christoffel Dinant, Jiri Bartek, Petra Hamerlik

**Affiliations:** 1 Brain Tumor Biology, Danish Cancer Society Research Center, Strandboulevarden 49, Copenhagen, DK-2100 Denmark; 2 Biotech Research and Innovation Centre, Faculty of Health and Medical Sciences, University of Copenhagen, Ole Maaløes Vej 5, Copenhagen, DK-2200 Denmark; 3 Department of Computational and RNA Biology, Department of Biology, University of Copenhagen, Ole Maaløes Vej 5, Copenhagen, DK-2200 Denmark; 4 Department of Neurosurgery, Copenhagen University Hospital, Blegdamsvej 9, Copenhagen, DK-2100 Denmark; 5 Department of Clinical and Molecular Pathology, Faculty of Medicine and Dentistry, Palacky University, Hnevotinska 3, Olomouc, 77515 Czech Republic; 6 University Hospital Olomouc, Hnevotinska 3, Olomouc, 77515 Czech Republic; 7 Genome Integrity Unit, Danish Cancer Society Research Center, Strandboulevarden 49, Copenhagen, DK-2100 Denmark; 8 Division of Genome Biology, Department of Medical Biochemistry and Biophysics, Science for Life Laboratory, Karolinska Institute, Scheeles vag 2, Stockholm, 17177 Sweden; 9 Department of Drug Design and Pharmacology, Copenhagen University, Jagtvej 160, Copenhagen, DK-2100 Denmark

**Keywords:** Cancer stem cells, Homologous recombination

## Abstract

Glioblastoma cancer-stem like cells (GSCs) display marked resistance to ionizing radiation (IR), a standard of care for glioblastoma patients. Mechanisms underpinning radio-resistance of GSCs remain largely unknown. Chromatin state and the accessibility of DNA lesions to DNA repair machineries are crucial for the maintenance of genomic stability. Understanding the functional impact of chromatin remodeling on DNA repair in GSCs may lay the foundation for advancing the efficacy of radio-sensitizing therapies. Here, we present the results of a high-content siRNA microscopy screen, revealing the transcriptional elongation factor SPT6 to be critical for the genomic stability and self-renewal of GSCs. Mechanistically, SPT6 transcriptionally up-regulates BRCA1 and thereby drives an error-free DNA repair in GSCs. SPT6 loss impairs the self-renewal, genomic stability and tumor initiating capacity of GSCs. Collectively, our results provide mechanistic insights into how SPT6 regulates DNA repair and identify SPT6 as a putative therapeutic target in glioblastoma.

## Abstract (web summary)

Cancer stem cells can evade treatment. Here, the authors perform an in vitro screen to identify proteins that are involved in protecting glioma cancer stem cells from therapy and find that SPT6 increases BRCA1 expression and drives error-free DNA repair, thereby ensuring the survival of the cells.

## Introduction

The integrity of mammalian genomes is continuously challenged by DNA double-strand breaks (DSBs), cytotoxic lesions that can be generated by cell-intrinsic processes, including DNA replication, transcription, and metabolism, resulting in an oxidative and replication stress. Thus, a functional DNA damage response (DDR) promoting DNA repair and cell cycle checkpoints is crucial for cellular survival^1^. The DDR encompasses a network of proteins that sense and respond to DSB formation. The failure or error-prone repair of DSBs can lead to cell death or accumulation of deleterious gross chromosomal aberrations, such as deletions, translocations, and fusions that promote genomic instability and tumorigenesis^2^. Rampant genomic instability and marked resistance to DNA damaging therapies are among the hallmarks of glioblastoma (GBM), one of the deadliest of solid tumors with median survival rates of ~15 months^3^. The causes of therapeutic resistance are diverse, but hierarchical organization of this disease with therapeutically resistant cancer stem-like cells (CSCs) at the apex is assumed a plausible cause of treatment failure ultimately leading to tumor recurrence^4^. Previously, we and others have demonstrated that gliomas, in general, and GBM-derived cancer stem-like cells (GSCs), in particular, display constitutive DDR activation as a consequence of continuous exposure to replication and oxidative stress^5–11^.

Eukaryotic cells have developed two major mechanisms for DSB repair: error-free homologous recombination (HR) and error-prone non-homologous end-joining (NHEJ)^12^. The choice of DSB repair is dictated by the antagonistic relationship of p53-binding protein 1 (53BP1) and breast cancer susceptibility protein type 1 (BRCA1) in the context of cell cycle stages. BRCA1 promotes HR by activating end-resection, which occurs in the S and G2 phases of the cell cycle, when sister chromatids are available. NHEJ prevails in G1 and is controlled by 53BP1, which acts as a barrier to HR by inhibiting DNA-end resection. Consequently, cells lacking BRCA1 display aberrant activation of NHEJ at replication-associated DSBs in S/G2 cell cycle phase, leading to gross chromosomal rearrangements^13–15^. Mouse studies have shown that 53BP1 loss causes growth retardation, immunodeficiency and radio-sensitivity, while preventing the chromosomal aberrations seen in BRCA1 null animals, stressing the importance of BRCA1-dependent removal of 53BP1 to mediate the transition from NHEJ to HR^14,16,17^.

Accumulating evidence suggests a crucial role of the chromatin state in DNA replication, transcription, repair and genomic stability of human cells. Chromatin compaction represents a major constraint limiting the timely access of DNA repair factors, which is crucial for efficient recognition and removal of DNA lesions^18^. Previously, we reported that BRCA1, a TF and chromatin remodeler, mediates responses of GBM cells to supra-physiological replications stress^9^, an inherent feature of this deadly disease^6^. Supporting the clinical relevance of chromatin regulation, several chromatin remodeling factors were identified as essential for GSC maintenance, thus opening a novel avenue for therapeutic intervention in GBM^19,20^.

Based on this background, we hypothesized that GSCs and the differentiated GBM cells (DGCs) differ in their dependency on chromatin remodeling genes to enable efficient DNA repair, which confers the superior capacity of GSCs to evade DNA damaging therapies. Using matched patient-derived GSCs and DGCs, we carried out a high-content siRNA screen, which identified the histone chaperone and transcription elongation factor SPT6 (Suppressor of Ty 6 homolog) as a key regulator of error-free DNA repair in GSCs. Moreover, SPT6 drives a stem cell transcriptional program to promote GSC maintenance and tumorigenicity in vivo, thus supporting SPT6 as a novel therapeutic target in GBM.

## Results

### siRNA screen identifies a role for SPT6 in DNA repair of GSC

**Fig. 1:**
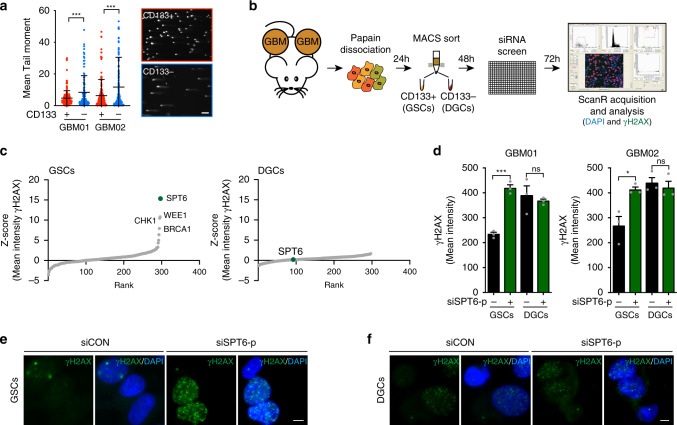
High-throughput siRNA screen identifies SPT6 as a regulator of genomic stability in glioblastoma stem-like cells. **a** DSBs quantification using a comet assay in matched GSCs and DGCs isolated from GBM01 and GBM02 GBM lines. Data are presented as mean values ± s.d. ****p < 0.0001 (GBM01), ***p = 0.0003 (GBM02); two-tailed unpaired *t*-test. The right part of the panel is showing representative images of comets captured using 10x objective. Scale bar = 100 μm. **b** Schematic illustration of siRNA microscopy-based screen work flow. **c** A scatter plot of z- scores derived from the siRNA screen for inducers of DSBs in matched GSCs and DGCs. The position of the three high-scoring hits with a reported role in DNA repair (WEE1 and CHK1) and our positive control (BRCA1) are indicated. The top-scoring hit, SPT6, is highlighted as a green dot. **d** γH2AX mean intensity quantification in matched GSCs and DGCs (GBM01 and GBM02) transfected with siCON or a pool of three independent siRNAs (siSPT6-p). Data are presented as mean values ± s.e.m. ***p = 0.0004 (GBM01-GSCs), non-significant ns = 0.637 (GM01-DGCs), *p = 0.022 (GBM02-GSCs), non-significant ns = 0.631 (GBM02-DGCs); two-tailed unpaired *t*-test. **e**, **f** Representative confocal microscopy images of γH2AX staining (green foci) in SPT6 silenced (siSPT6-p) GSCs (**e**) and DGCs (**f**) compared to respective control (siCON). Nuclei were counterstained with DAPI (blue). Images were captured using ×40 objective. Scale bar = 5 μm. *N* = 3 biologically independent experiments in (**a**), (**d**), (**e**), (**f**). Source data are provided as a Source data file.

DNA damage poses a serious threat to cell survival by compromising both genomic and epigenomic integrity. DDR activation is accompanied by chromatin remodeling, affecting intrinsic chromatin components and epigenetic marks, thereby assuring the accessibility of DNA lesions and timely recruitment of DNA repair machinery^21^. At baseline, the GSCs accumulate a significantly lower amount of DSBs (Fig. 1a) compared to DGCs. This observation together with previously reported dependency of GSCs on chromatin remodeling factors^19,22^ prompted us to interrogate the impact of the chromatin state on DNA repair efficiency of matched GSC and DGC sub-populations. We performed a microscopy-based screen using a siRNA library covering 296 chromatin remodeling genes. Candidates were selected whose knockdown augmented DSBs, which we assessed by scoring γH2AX mean intensity (MI) (Fig. 1b and Supplementary Data 1). Z-score was calculated for γH2AX MI in matched patient-derived GSCs and DGCs (Supplementary Data 2 and Fig. 1c) and candidates were selected that induced γH2AX MI (≥20% fold change over the mean of scrambled controls) in GSCs but not in DGCs. Factors such as WEE1, CHK1, and BRCA1, with an already established role in DNA repair, ranked among the top hits. The largest induction of γH2AX MI was seen upon downregulation of the Suppressor of Ty 6 homolog (SPT6), a histone chaperon that binds the C-terminal repeat domain (CTD) of RNAP II via its tandem SH2 domain^23–26^, and is essential for transcription elongation of RNAP II-transcribed genes^27^. Further validation experiments using a pool of three independent siRNAs targeting SPT6 (siSPT6-p) as well as individual siRNAs (siSPT6-34; siSPT6-35; siSPT6-36) confirmed that SPT6 loss-mediated DSBs induction is unique to GSCs (assessed by γH2AX MI, γH2AX foci count quantification or immunoblot; Fig. 1d–f; Supplementary Fig. 1).

### SPT6 is essential for GSC maintenance

**Fig. 2:**
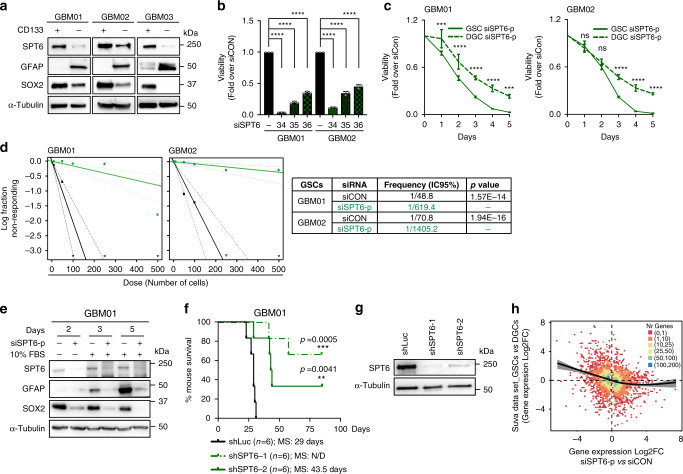
SPT6 as a regulator of genomic stability in GSCs. **a** Representative immunoblot analysis of SPT6, GFAP and SOX2 expression in GSCs and DGCs in GBM01, GBM02 and GBM03 lines. **b** Viability assay of GSCs (GBM01, GBM02) transfected with siCON or siRNAs targeting siSPT6 (siSPT6-34; siSPT6-35; siSPT6-36. Data are presented as mean values ± s.d. ****p < 0.0001; two-way ANOVA analysis with Sidak’s multiple comparisons test. **c** Viability assay of matched GSCs and DGCs (GBM01; GBM02) transfected with siCON or siSPT6-p. Data are presented as mean values ± s.d and normalized to siCON. GBM01: ***p = 0.005 (1 day), ****p < 0.0001 (day 2, 3, 4), ***p = 0.0001 (day 5); GBM02: non-significant *p* = 0.866 and *p* = 0.192 (day 1, 2, respectively), ****p < 0.0001 (3, 4, 5 days); two-way ANOVA and Sidak’s multiple comparisons test. (**d**) Representative extreme limiting dilution assay (ELDA) of GSCs (GBM01 and GBM02) transfected with siCON or siSPT6-p. Respective *p*-values (GBM01 *p* = 1.57E-14; GBM02 *p* = 1.94E-16 by ELDA analysis program) are listed in a table summary. **e** Representative immunoblot analysis of SPT6, GFAP, and SOX2 expression in GSCs transfected with siCON or siSPT6-p undergoing serum-(FBS)-induced differentiation. (**f**) Kaplan–Meier survival curves of mice implanted with GBM01 GSCs with silenced SPT6 (shSPT6-1 and shSPT6-2) compared to control (shLuc). *n* = 6 mice in each arm. **p = 0.0041; ***p = 0.0005; Log-rank/Mantel-Cox test. MS median survival, ND non-determined. **g** Representative immunoblot validation of SPT6 knockdown efficiency in GSCs used in (**f**). **h** Spearman correlation analysis of SPT6 expression and stem cell transcriptional signature using the Suva data set^28^. *r*_s_: −0.281, *p*-value < 2.2e-16. *X*-axis: positive values upregulated after SPT6 loss. *Y*-axis: positive values upregulated in GSCs cells. Color indicates the number of genes in each region. The black trend line is calculated as a smoothed average along the *X*-axis where the shaded region indicates standard error. *N* = 3 biologically independent experiments in (**a**–**e**), (**g**). Loading control: α-Tubulin in (**a**), (**e**), (**g**). Source data are provided as a Source data file.

Based on the selective induction of DSBs upon SPT6 silencing in GSCs, we measured the relative expression of SPT6 in three matched pairs of GSCs and DGCs. On immunoblot, GSCs preferentially expressed SPT6 protein relative to DGCs (Fig. 2a). The difference in SPT6 protein levels translated to a higher dependency of GSCs on SPT6 expression in vitro, as its silencing reduced the viability of GSCs to a greater extent than that of DGCs, impaired their self-renewal as well as differentiation potential (Fig. 2b–e and Supplementary Fig. 2a–b). One of the key properties of GSCs is the capacity to initiate tumors in immunocompromised hosts. GSCs transduced with either shRNA targeting luciferase (shLuc) or two independent shRNAs targeting SPT6 (shSPT6-1 and shSPT6-2) were implanted orthotopically into immunocompromised mice. SPT6 loss extended the median survival of tumor-bearing mice from 29 (shLuc) to 43.5 days for shSPT6-2 (*p* = 0.0041). The median survival for shSPT6-1 group was not reached as four animals remained alive at the end of study (Fig. 2f, g; for the impact of shRNA-mediated knockdown using shSPT6-1 and shSPT6-2 on the viability of GSCs in vitro, see Supplementary Fig. 2c–d). Altogether, these findings validate SPT6 as a regulator of CSCs’ maintenance.

To determine the molecular mediators of SPT6 function in GSCs, we performed differential RNA analysis (further referred to as the SPT6 data set) analysis of GSCs transfected with control siRNA (siCON) or siRNA targeting SPT6 (siSPT6-p) (Supplementary Data 3). Supportive of the inherent dependency of GSCs on SPT6 expression, gene set enrichment analysis revealed significant downregulation of gene sets involved in stem cell maintenance and differentiation (Supplementary Fig. 2e, Supplementary Data 4). To further validate our findings, we leveraged the Suva data set covering differential expression profiles of three matched GSCs and DGCs^28^. A Spearman correlation analysis confirmed positive association between the SPT6 knockdown-associated transcriptional changes and the transcriptional program of GSCs in SUVA data set (Fig. 2h, Supplementary Fig. 2e and Supplementary Data 5; false discovery rate, FDR < 0.05; *r*_s_: −0.281, *p*-value < 2.2e−16).

### SPT6 silencing induces DDR, cell cycle arrest, and apoptosis

**Fig. 3:**
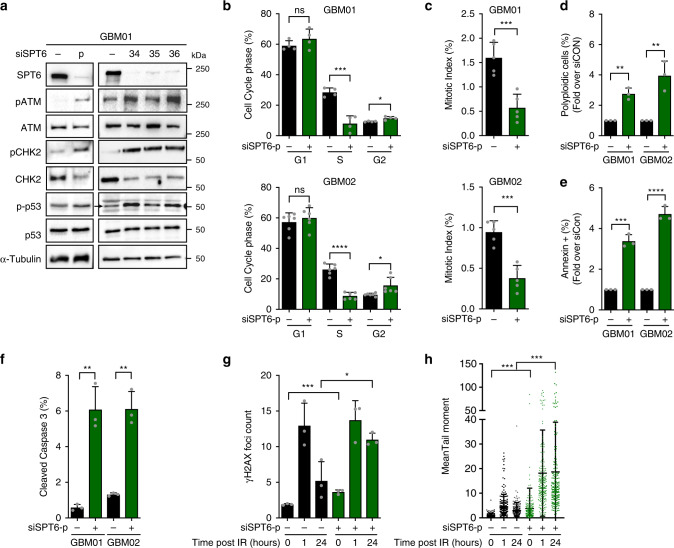
SPT6 loss activates DDR activation, cell cycle arrest, and apoptosis. **a** Representative immunoblot analysis total and phosphorylated ATM, CHK2, and p53 in GSCs (GBM01) transfected with siCON, siSPT6-p or three independent siRNAs targeting siSPT6 (siSPT6-34; siSPT6-35; siSPT6-36). Loading control: α-Tubulin. **b** FACS analysis of cell cycle profile in GSCs (GBM01 and GBM02) transfected with siCON or siSPT6-p. Bar graph indicates the % of cells in G1, S and G2. Data are presented as mean values ± s.d. GBM01: non-significant ns = 0.254, ***p = 0.0004, **p = 0.0077, GBM02: non-significant *p* = 0.477, ****p < 0.0001, *p = 0.0207. **c** Mitotic index (MI; % of H3Ser10-positive cells) was assessed by FACS analysis of GSCs transfected with siCON or SPT6-p (GBM01 and GBM02). Data are presented as mean values ± s.d. ***p = 0.006 (GBM01), ***p = 0.0003 (GBM02). **d** FACS analysis of polyploid cells fraction (%) in GSCs transfected with siCON or siSPT6-p. Data are presented as mean values ± s.d. **p = 0.0013 (GBM01), **p = 0.0062 (GBM02). **e** FACS-based quantification of Annexin V-positive (%) GSCs after transfection with siCON or siSPT6-p. Data are presented as mean values ± s.d. ***p = 0.0002 (GBM01), ***p < 0.0001 (GBM02). **f** FACS analysis of cleaved caspase-3-positive GSCs after transfection with siCON or siSPT6-p. Data are presented as mean values ± s.d. ***p = 0.0018 (GBM01), **p = 0.0011 (GBM02). **g** γH2AX foci quantification at 0, 1 and 24 hrs after the exposure to ionizing radiation (IR, 3 Gy) in GSCs transfected with siCON or siSPT6-p. Data are presented as mean ± s.d. ***p = 0.0007, *p = 0.0101. **h** Representative DSBs quantification using comet assay (**h**) 0, 1, and 24 h after the exposure to ionizing radiation (IR, 3 Gy) in GSCs transfected with siCON or siSPT6-p. Data are presented as mean  ± s.d. ****p < 0.001. *N* = 3 biologically independent experiments in (**a**), (**d**–**h**), *N* = 4 biologically independent experiments in (**b**) GBM01, *N* = 5 biologically independent experiments in (**c**) and *N* = 6 biologically independent experiments in (**b**) GBM02. Two-tailed unpaired *t*-test used in (**b**–**h**). Source data are provided as a Source data file.

Activation of the DDR coordinates the inherent response of cells to DNA damage, in which ATM and ATR kinases monitor genome integrity to activate the DDR^29^. Activated ATM leads to phosphorylation of γH2AX, and the activation of mediators and adaptor proteins, such as 53BP1 and BRCA1^14,30^. Immunoblot analysis to investigate the effects of SPT6 silencing on the DDR revealed increased phosphorylation of ATM. ATM activates the cell-cycle checkpoints or induces apoptosis by phosphorylating itself and the effector kinase CHK2, which in turn activates the tumor suppressor p53 through Ser15 phosphorylation^31^. SPT6 silencing induced the activating phosphorylation of CHK2 at Thr68 and p53 at Ser15, thus confirming DDR activation (Fig. 3a). Next, we examined the cell cycle kinetics in GSCs transfected with siCON and siSPT6. As shown in Fig. 3b, c (and Supplementary Fig. 3a–b), SPT6 knockdown decreased both the proliferative (the percentage of Ethynyl deoxyUridine positive S phase cells) and mitotic (the percentage of H3Ser10 positive mitotic cells) indexes and arrested GSCs at G2 phase. Aligned with the impaired DNA repair capacity and DSB accumulation, SPT6 knockdown markedly increased the number of polyploid cells and the level of apoptosis (assessed by FACS analysis of Annexin V and cleaved caspase 3 stained GSCs; Fig. 3d–f and Supplementary Fig. 3c–d).

The preferential DDR activation in GSCs is thought to be a pre-requisite for their radio-resistance^5^. Thus, we sought to test the significance of SPT6 in the IR-induced DNA damage repair capacity of radio-resistant GSCs. Quantification of IR-induced γH2AX foci as well as measuring the tail moments with a comet assay, confirmed significantly delayed DSB repair kinetics of GSCs with silenced SPT6 compared to siCON (Fig. 3g, h and Supplementary Fig. 3e–f). Collectively, these data suggest that SPT6 is essential for DSBs repair, cell cycle progression and contributes to radiation resistance in GBM.

### SPT6 regulates the transcription of HR repair genes

**Fig. 4:**
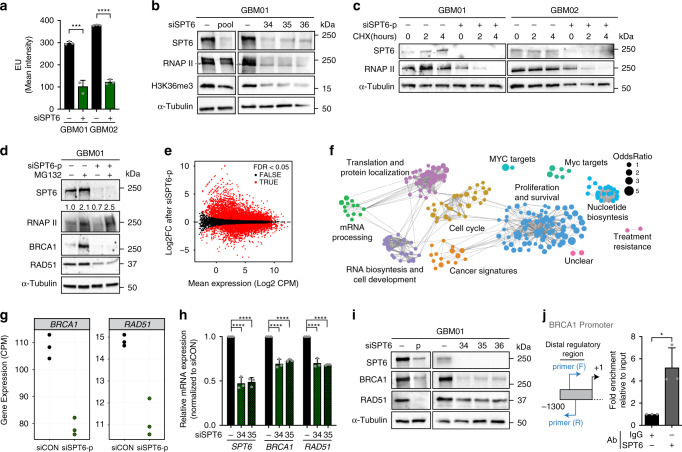
SPT6 as a transcriptional regulator of HR gene expression. **a** FACS analysis of global transcription in GSCs transfected with siCON or siSPT6-p. Data shown as mean ± s.d. ***p = 0.0003, ****p < 0.001; two-tailed unpaired *t*-test. **b** Representative immunoblot analysis of SPT6, RNAP II and H3K36me3 levels in GSCs transfected with siCON, siSPT6-p or siSPT6-34, siSPT6-35 or siSPT6-36. **c**Representative immunoblot analysis of SPT6 and RNAP II levels in GSCs transfected with siCON or siSPT6-p and exposed to cycloheximide (CHX; 20 µg ml^−1^) at 0, 2 or 4 h. **d** Representative immunoblot analysis of SPT6, RNAP II, BRCA1 and RAD51 levels in GSCs transfected with siCON or siSPT6-p followed by MG132 treatment for 5 h (20 µM). **e** MA plot depicting the extend of transcriptional changes associated with SPT6 loss. Red dots indicate significantly differentially expressed genes. EdgeR function, FDR < 0.05. **f** Gene Set Enrichment Analysis (GSEA) showing significantly downregulated signaling pathways in GSCs with silenced SPT6 (siSPT6-p). The circle size specifies the enrichment (odds ratio) and the thickness of the connecting lines the overlap in-between gene sets. **g** *BRCA1* and *RAD51* are downregulated in GSCs with silenced SPT6. *p* = 2.6e-07 (*BRCA1*) and *p* = 1.8e−0.3 (*RAD51*) edgeR function. **h** qRT-PCR analysis of *SPT6, BRCA1 and RAD51* expression in GSCs with silenced SPT6 (siSPT6-34 and siSPT6-35). House-keeping gene control: HPRT. Data are normalized to siCON and presented as mean ± s.d. ****p < 0.001; two-way ANOVA followed by Dunnett’s multiple comparison test. **i** Representative immunoblot analysis of SPT6, BRCA1 and RAD51 expression in GSCs transfected with siCON or siSPT6-p. **j** Left: Schematic representation of BRCA1 promoter primer sites. Right: Chromatin immunoprecipitation of SPT6 followed by qRT-PCR in GSCs. Bar graph shows relative fold enrichment compared to input (10%). Data are presented as mean ± s.d. *p = 0.0148; two-tailed unpaired *t*-test. *N* = 3 biological independent experiment in (**a**–**d**), (**h**–**j**). Loading control: α-Tubulin in (**b**–**d**), (**i**). Source data are provided as a Source data file.

In immunofluorescence staining, SPT6 did not co-localize with γH2AX foci (Supplementary Fig. 4a), implying that its impact on DNA repair may be indirect. The role of SPT6 as a transcription elongation factor prompted us to speculate that SPT6 executes its role in DSB repair by modulating the transcription of RNA polymerase II (RNAP II)-transcribed genes, in general, and DNA repair genes, in particular. First, we assessed the impact of SPT6 loss on RNAP II protein levels and global transcription rates (assessed by pulse-labeling of nascent RNA with Ethynyl Uridine; EU followed by FACS). Global transcription rates declined by more than 50% upon SPT6 downregulation (Fig. 4a and Supplementary Fig. 4b), a phenomenon associated with a marked decrease in RNAP II and H3K36me3 levels (Fig. 4b and Supplementary Fig. 4c). Cycloheximide (CHX) chase assay showed a significantly decreased half-life of RNAP II in cells with silenced SPT6 (Fig. 4c). When treated with proteasomal inhibitor MG132 (20 µM, 5 h), RNAP II protein levels in GSCs transfected with siRNA targeting SPT6 increased (Fig. 4d). Overall, these data suggest that in GSCs, SPT6 binding to RNAP II prevents its ubiquitin-mediated proteasomal degradation and thereby maintains the global transcription rates.

To gain deeper insight into the mechanism(s) contributing to the SPT6-mediated DNA repair and cell proliferation, we leveraged the SPT6 data set and searched for genes the transcription of which was significantly altered as a result of SPT6 loss. As represented by Bland-Altman (MA plot) plot, 4966 genes were significantly upregulated and 4969 genes were downregulated in GSCs with silenced SPT6 (FDR < 0.05; Fig. 4e and Supplementary Data 3 and 4). An unbiased clustering analysis identified SPT6 as a regulator of RNA metabolism, cell survival, proliferation and carcinogenesis (Fig. 4f and Supplementary Data 6). Gene Set Enrichment Analysis (GSEA) indicated that DNA repair transcriptional programs were downregulated in GSCs with SPT6 knockdown (FDR < 0.05; Supplementary Fig. 4d and Supplementary Data 4), an observation mirroring the impaired capacity of GSCs to repair DSBs upon SPT6 loss. *BRCA1* and *RAD51* (*p* = 2.6e−07 and *p* = 1.8e−03, respectively), two factors essential for DSBs repair via error-free HR repair (further denoted as ‘HR genes’) were among significantly downregulated DNA repair genes in SPT6 data set. A validation experiment using qRT-PCR confirmed that the SPT6 knockdown-associated reduction in *BRCA1* and *RAD51* levels is unique to GSCs (Fig. 4g, h and Supplementary Fig. 4e). Consistent with a decrease in mRNA expression, immunoblot analysis confirmed lowered BRCA1 and RAD51 protein levels in GSCs with silenced SPT6 (Fig. 4i and Supplementary Fig. 4c).

SPT6 knockdown-mediated decline in RNAP II protein and global transcription (see above), could contribute to such a decrease in *BRCA1*/*RAD51* expression. Thus, we examined the correlation between transcriptional changes observed upon SPT6 knockdown and previously reported half-life of human coding mRNAs^32^. This analysis confirmed that our results are not biased by the global decrease in transcription and RNAP II protein levels (Supplementary Fig. 4f).

While MG132 treatment restored RNAP II protein levels, both mRNA and protein levels of BRCA1 and RAD51 remained lower in GSCs with silenced SPT6 (Fig. 4d and Supplementary Fig. 4g), implying that SPT6 is essential for RNAP II-mediated transcription of *BRCA1* and *RAD51*. To validate that SPT6 acts as a transcriptional co-activator of *BRCA1* expression in GSCs, we performed a ChIP-qPCR assay. As shown in Fig. 4j, this assay confirmed SPT6 binding to distal regulatory region of *BRCA1* gene promoter.

### SPT6 dictates DSB repair pathway choice

**Fig. 5:**
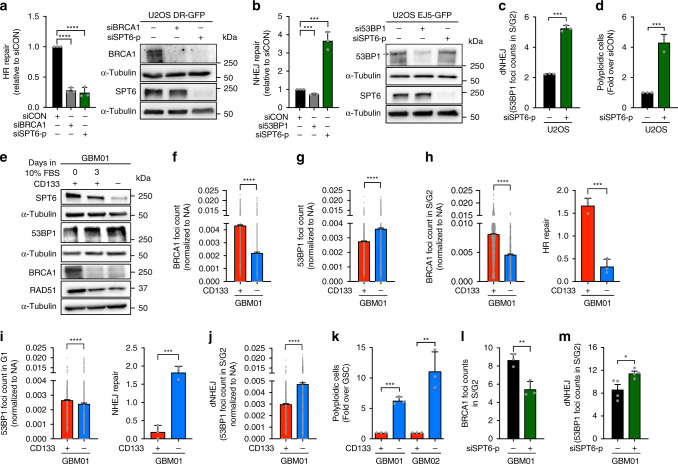
SPT6 levels dictate DSBs repair pathway choice. **a** Left: HR repair in DR-GFP cells transfected with siCON, siSPT6-p or siBRCA1. Data presented as mean ± s.d. ****p < 0.0001. Right: Representative immunoblot of BRCA1 and SPT6 levels in DR-GFP cells transfected with siCON, siSPT6-p or siBRCA1. **b** Left: NHEJ repair in EJ5-GFP cells transfected with siCON, siSPT6-p or si53BP1. Data presented as mean ± s.d. ***p = 0.0005 and ***p = 0.0007. Right: Representative immunoblot of 53BP1 and SPT6 levels in EJ5-GFP cells transfected with siCON, siSPT6-p, si53BP1. **c** dNHEJ in U2OS cells transfected with siCON or siSPT6-p. Data are presented as mean ± s.d. ****p < 0.0001. **d** FACS analysis of polyploid cells after SPT6 silencing in U2OS. Data normalized to siCON and presented as mean ± s.d. ***p = 0.0004. **e** Representative immunoblot analysis of SPT6, 53BP1, BRCA1 and RAD51 expression in GSCs, differentiated GSCs and DGCs. **f** BRCA1 foci quantification in matched GSCs (n = 8505) and DGCs (*n* = 2690). ****p < 0.0001. (**g**) 53BP1 foci quantification in matched GSCs (*n* = 9704) and DGCs (*n* = 5817). ****p < 0.0001. **h** Left: BRCA1 foci quantification in S/G2 phase GSCs (*n* = 3511) and DGCs (*n* = 1011). ****p < 0.0001. Right: HR repair in matched GSCs and DGCs. Data are presented as mean ± s.d. ***p = 0.0005. **i** Left: 53BP1 foci quantification in G1 phase GSCs (*n* = 5403) and DGCs (*n* = 2473). ****p < 0.0001. Right: NHEJ repair in matched GSCs and DGCs. Data are presented as mean ± s.d. ***p = 0.0003. **j** dNHEJ in matched GSCs (*n* = 3451) and DGCs (*n* = 1802). ****p < 0.0001. **k** FACS analysis of polyploid cells in matched GSCs and DGCs. Data presented as mean ± s.d. ***p = 0.0001 and **p = 0.0054. **l** BRCA1 foci quantification in S/G2 phase GSCs transfected with siCON or siSPT6-p. Data are presented as mean ± s.d. **p = 0.0068. **m** dNHEJ in GSCs transfected with siCON or siSPT6-p. Data are presented as mean ± s.d. *p = 0.0273. *N* = 3 biologically independent experiments in (**a**–**m**). Loading control: α-Tubulin in (**a**), (**b**), (**e**). The statistical test used in (**a**–m): two-tailed unpaired *t*-test. Data from cells examined over three biologically independent experiments (*n* = number of cells) are normalized to Nuclear Area (NA) and presented as mean ± s.e.m. in (**f**), (**g**), (**h**) Left, (**i**) Left, (**j**). Source data are provided as a Source data file.

To evaluate the extent of HR suppression after SPT6/BRCA1 loss, we employed the well-established DR-GFP assay using U2OS cells (a benchmark cell line widely used in DNA repair field to measure HR activity). SPT6 knockdown in U2OS cells reproduced the DNA repair phenotype observed in GSCs (Supplementary Fig. 5). siRNAs targeting SPT6 resulted in HR suppression similar in magnitude to that seen with siRNAs targeting the key HR protein BRCA1 (Fig. 5a). BRCA1 was reported to antagonize 53BP1-dependent DNA repair in S/G2 phase by inhibiting its interaction with chromatin proximal to DSB^33^. The activation of error-prone repair of DSBs by NHEJ (here referred to as de-regulated NHEJ; dNHEJ) increases the frequency of deleterious mutagenic events^34^ and is more prevalent in polyploid cancer cells^35^. Besides a significant increase in NHEJ (Fig. 5b), SPT6 knockdown increased the frequency of dNHEJ (evaluated by scoring 53BP1 foci count in S/G2 cells) as well as the number of polyploid cells (Fig. 5c, d).

Similar to SPT6, HR genes were preferentially expressed by GSCs and their expression decreased during serum-induced differentiation. 53BP1 expression (NHEJ) followed the opposite trend, with both the total protein levels as well as foci count increased in DGCs (Fig. 5e–g). To examine the impact of differential BRCA1 and 53BP1 expression on the DSBs repair pathway choice in the context of cellular hierarchies, we have interrogated HR and NHEJ by scoring the BRCA1/53BP1 foci formation and conducted DR-GFP/pEGFP-N3 plasmid-based assay in a matched pair of GSCs and DGCs. This analyses revealed that GSCs primarily utilize HR, which is concordant with elevated BRCA1 recruitment in S/G2 phase cells (Fig. 5h). Even though 53BP1 recruitment (foci count) was higher in G1 phase GSCs, the pEGFP-N3 plasmid-based repair assay confirmed higher NHEJ rates in DGCs (Fig. 5i). Given that DGCs expressed much lower levels of BRCA1, we speculated that this is due to the elevated 53BP1-dependent DNA repair in S/G2 phase (dNHEJ). Indeed, microscopy-based analysis of 53BP1 foci count (in the context of cell cycle) revealed that dNHEJ rate in DGCs is markedly increased compared to that of GSCs (Fig. 5j), and associates with a significant increase in the frequency of polyploid cells (Fig. 5k). Importantly, SPT6 knockdown reduced BRCA1 expression and HR activity in GSCs, and shifted DSBs repair towards dNHEJ (Fig. 5l, m and Supplementary Fig. 6a–c). Altogether, these data indicate that the SPT6 ^high^ /BRCA1^high^-expressing GSCs execute DSBs repair predominantly via HR to maintain genomic stability, while SPT6 ^low^ /BRCA1^low^-expressing DGCs rely on NHEJ/dNHEJ, which renders them more susceptible to DSBs formation and genomic instability.

### Ectopic BRCA1 expression rescues SPT6 loss phenotype

**Fig. 6:**
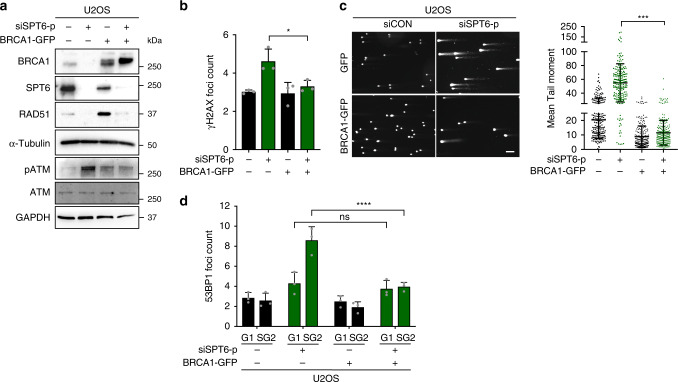
Ectopic BRCA1 expression rescues SPT6 loss phenotype. **a** Representative immunoblot analysis of BRCA1, SPT6, RAD51, total and phosphorylated ATM expression in U2OS-GFP and U2OS-BRCA1-GFP cells transfected with siCON or siSPT6-p (pool). Loading control: α-Tubulin and GAPDH. **b** γH2AX foci quantification in U2OS-GFP and U2OS-BRCA1-GFP cells transfected with either siCON or siSPT6-p. Data are presented as mean ± s.d. *p = 0.0331; one-way ANOVA followed by Tukey’s multiple comparison test. **c** Representative DSBs quantification using comet assay. Data are presented as mean ± s.d. ****p < 0.0001; one-way ANOVA followed by Tukey’s multiple comparison test. Representative images are shown on left. **d** 53BP1 foci quantification in G1 and S/G2 phase U2OS-GFP and U2OS BRCA1-GFP cells transfected with siCON or siSPT6-p. Non-significant ns = 0.8471 and ****p < 0.0001; two-way ANOVA followed by Tukey’s multiple comparison test. *N* = 3 biologically independent experiments in (**a**–**d**). Source data are provided as a Source data file.

Stable binding of 53BP1 to DNA ends channels DSB repair into NHEJ, in part by suppressing end resection, a process that is necessary for the generation of long stretches of single-stranded DNA (ssDNA) needed for HR^36,37^. In the absence of BRCA1, 53BP1 has been shown to promote mutagenic NHEJ at replication-associated DSBs, leading to gross chromosomal rearrangements^14^. To understand whether BRCA1 loss is responsible for the activation of dNHEJ, and whether its ectopic expression rescues the SPT6-BRCA1 loss phenotype, we stably overexpressed BRCA1 (BRCA1-GFP) in U2OS cells (Fig. 6a). BRCA1-GFP cells and cells transfected with backbone control (GFP) were then transfected with either siCON or siSPT6. The introduction of ectopic BRCA1 rescued the activation of DDR (assessed by immunoblot analysis of ATM Ser1981 phosphorylation) upon SPT6 silencing (Fig. 6a) as well as DSB induction as assessed by γH2AX foci count and comet assay (Fig. 6b, c). Importantly, re-introduction of BRCA1 prevented 53BP1 recruitment to DSBs in S/G2 and restricted 53BP1 foci formation to G1 cell cycle phase (Fig. 6d).

### Chaetocin as a drug candidate mimicking ‘SPT6 LOSS’

**Fig. 7:**
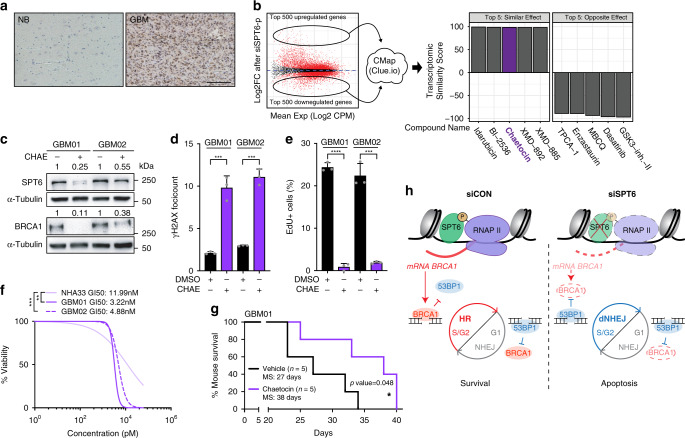
CMap identifies chaetocin as a drug candidate mimicking SPT6 loss in GSCs. **a** Representative IHC staining of SPT6 in normal brain (NB; *n* = 10) and GBM (*n* = 24). Scale bar 100 μm. **b** Schematic representation of the Connectivity Map (CMap) analysis. Top 500 up- and downregulated genes after SPT6-p (pool) silencing was extracted and uploaded to CMap, which generated a list of chemical compounds mimicking SPT6 loss at transcriptional level. **c** Representative immunoblot analysis of SPT6 and BRCA1 protein levels in GSCs (GBM01 and GBM02) treated with chaetocin (CHAE; 24 h of 30 nM). Loading control: α-Tubulin. **d** γH2AX foci quantification 24 h after exposure to chaetocin (CHAE, 30 nM) in GSCs (GBM01 and GBM02). Data are presented as mean ± s.d. ***p = 0.0007 (GBM01) and ***p = 0.0001; two-tailed unpaired *t*-test. **e** Quantification of EdU positive cells (%) after 24 h after the treatment with chaetocin (CHAE; 30 nM) in GSCs (GBM01 and GBM02). Data are presented as mean ± s.d. ****p < 0.0001 (GBM01) and ***p = 0.0002 (GBM02); two-tailed unpaired *t*-test. **f** GI_50_ calculation CHAE treatment in GSCs (GBM01 and GBM02) and NHA33 cells. **p = 0.0017 (NHA33 *vs* GBM01) and ***p = 0.0010 (NHA33 *vs* GBM02); one-way ANOVA followed by Dunnett’s multiple comparison test. **g** Kaplan–Meier survival curves for mice implanted with GBM01 GSCs and treated intraperitoneally with either vehicle (DMSO) or chaetocin (*n* = 5 mice per group) three times a week for a period of two weeks. MS = Median Survival. Statistical significance was tested using Log-rank/Mantel-Cox test; *p = 0.048. **h** Model depicting the mechanistic role of SPT6 in the regulation of DSB repair pathway choice via the transcriptional regulation of *BRCA1*. *N* = 3 biologically independent experiments in (**c**–**f**). Source data are provided as a Source data file.

One of the first criteria to consider SPT6 a putative therapeutic target, SPT6 expression would be expected higher in gliomas compared to that of normal brain (NB) to rule out potential side effects of its targeting in healthy tissue. Indeed, immunohistochemistry (IHC) analysis revealed that the expression of SPT6 is elevated in GBM (*n* = 24) compared to non-malignant brain control (NB; *n* = 10) (Fig. 7a and Supplementary Fig. 7a). Immunoblot analysis confirmed higher expression of SPT6 in protein extracts from primary GBM patient-derived GSCs (GBM01-03) compared to normal human astrocytes (NHA33; NHA59) and whole-brain lysate (WBL; Supplementary Fig. 7b).

In silico analysis of the REMBRANDT data set showed that *SPT6* expression is significantly increased in malignant gliomas compared to NB and that this expression does not correlate with WHO grade (Supplementary Fig. 7c–d). Kaplan–Meier survival analysis confirmed a negative correlation between high *SPT6* expression (*SPT6*^high^) and glioma (WHO grade II–IV) patient survival (Supplementary Fig. 7e). When assessing the impact of *SPT6* expression on GBM (WHO grade IV) patient survival only (REMBRANDT data set), *SPT6*^low^ group exhibited longer survival compared to that of *SPT6*^high^ GBM patients (Supplementary Fig. 7f). Additional analysis of Gravendeel, TCGA GBM Array and TCGA GBM RNAseq data sets failed to validate these findings, concluding no correlation between *SPT6* expression and GBM patient survival (Supplementary Fig. 7g).

Due to the lack of specific inhibitor of SPT6 on the market, we leveraged an open resource CONNECTIVITY MAP (CMap)^38^ and matched the transcriptome profile of GSCs with silenced SPT6 to data sets available in CMap (Fig. 7b and Supplementary Data 7). This approach identified chaetocin, a non-specific inhibitor of histone lysine methyltransferases, as our top candidate mimicking SPT6 loss at a transcriptome level. As shown in Fig. 7c–e and Supplementary Fig. 7g, chaetocin treatment reduced both SPT6 and BRCA1 protein and mRNA levels, induced DSBs and reduced cell proliferation. Importantly, GSCs showed greater sensitivity to chaetocin than NHA (Fig. 7f), thus opening a therapeutic window for SPT6 targeting in GBM. Next, we evaluated the effect of chaetocin on tumor-initiating capacity of GSCs. Strikingly, the treatment of GSCs with as little as 30 nM of chaetocin completely abrogated their capacity to initiate tumors in vivo (Supplementary Fig. 7h). To further explore the therapeutic potential of chaetocin, we treated tumor-bearing mice intraperitoneally with a vehicle control (DMSO) or 1 mg kg^−1^ of chaetocin triweekly over a period of two weeks (total dose of 6 mg kg^−1^). As shown in Fig. 7g, chaetocin treatment significantly extended the survival of tumor-bearing mice by 11 days (*p*-value = 0.048).

Based on the data presented above, we propose a model (Fig. 7h), in which SPT6 acts as transcriptional co-activator of *BRCA1* expression in GSCs. BRCA1 then directs DSBs repair towards error-free repair by HR. Upon SPT6/BRCA1 loss, DNA repair pathway choice is deregulated and allows for the activation of dNHEJ, which increases the genomic instability of GSCs, ultimately leading to cell death by apoptosis. Both in vitro and in vivo studies using siRNA/shRNA targeting SPT6 and chaetocin as a drug mimicking the SPT6 loss phenotype validate SPT6 as a bona fide therapeutic target in GBM.

## Discussion

To identify new determinants governing the preferential capacity of GSCs for error-free DSBs repair, we performed a microscopy-based high-throughput siRNA screen, which revealed vulnerabilities specific to this therapeutically resistant sub-population of GBM. Importantly, proteins such as WEE1, CHK1 and BRCA1 with already established role in DNA repair, ranked among the top candidates. In response do DNA damage, WEE1 activates G2/M checkpoint. Its inhibition in GBM cells abrogated the G2/M arrest and propelled them to prematurely enter into mitosis and consequent cell death through mitotic catastrophe and apoptosis^39^. CHK1 is a key DDR factor, the loss of which leads to G2 checkpoint abrogation and sensitizes GSCs to ionizing radiation (IR)^40^. BRCA1 functions in a number of cellular pathways that maintain genomic stability, including DNA damage-induced cell cycle checkpoint activation, DNA repair, replication fork integrity, chromatin remodeling, as well as transcriptional regulation and apoptosis^41^. Recently, we have discovered a tumor-promoting role of BRCA1 in GBM, in supporting tumor cell fitness and growth through protection from endogenous replication stress via transcriptional upregulation of RRM2^9^.

Here, we identify SPT6, a histone chaperone and transcription elongation factor, to be essential for the DNA repair capacity and maintenance of GSCs. SPT6 plays a key role in the regulation of transcription initiation, elongation, and 3′ end formation^24,25,42–44^. Mutation of SPT6 facilitates chromatin relaxation in specific genomic regions, which impacts the regulation of transcription, genomic stability and hyperrecombination^23,43–48^. Previous reports implicated SPT6’s chromatin remodeling activities to regulate zebrafish myogenesis^49^ and mouse embryogenesis^50^. Here, we describe a pro-tumorigenic role for SPT6 in GBM and provide a mechanistic insight into its role in one of the deadliest of solid cancers. We find SPT6 overexpressed in GBM when compared to non-malignant brain controls (Fig. 7a). In silico analysis of REMBRANDT data set confirmed increased *SPT6* expression in malignant gliomas (compared to NB) to be negatively correlated with patients’ survival (Supplementary Fig. 7e–f). However, when assessing the impact of *SPT6* expression on the survival of GBM patients using additional data sets (Gravendeel, TCGA GBM array and TCGA GBM RNAseq, Supplementary Fig. 7g), we found no association between *SPT6* expression and GBM patient survival, implying that the prognostic value of *SPT6* is not independent of other variables such as WHO grade.

Spt6 depletion in mouse ESCs decreases expression of pluripotency transcription factors (TFs) and increases transcription of cell-lineage-affiliated master TFs, leading to morphological changes indicative of early cell differentiation^45^. Our data now show that GSCs preferentially express SPT6, which is not only essential for their maintenance and differentiation capacity, but also tumorigenicity in vivo (Fig. 2).

The integrity of the human genome is constantly endangered by a wide range of spontaneously occurring DNA lesions. BRCA1 and 53BP1 play a crucial role in regulating the DSB repair pathway choice throughout the cell cycle, channeling DSBs repair into either HR (S/G2 phase) or NHEJ (mainly G1 phase). Loss of BRCA1 was found to cause a shift towards the mutagenic NHEJ thereby inducing genomic instability, tumorigenesis, and embryonic lethality^16,36^. Proper DNA repair pathway choice is thereby paramount to cell survival, as the aberrant engagement of the NHEJ pathway following replication fork collapse leads to gross chromosomal rearrangements and cell death^51^. In 2006, Bao et al. reported that GSCs, which preferentially activate DDR, represent the radio-resistant sub-population in GBM tumor bulk. GSCs, in contrast to DGCs, exhibit superior survival by evading IR-induced apoptosis. Our findings show that at baseline, GSCs have fewer unrepaired DSBs, which can be attributed to their superior efficiency at executing DSB repair via both HR and NHEJ (Figs. 1a and 5h, i). In our model, the high SPT6-regulated BRCA1 expression in GSCs limits the recruitment of 53BP1 to DSBs in S/G2 phase cells and thus prevents dNHEJ (Fig. 7h). In contrast to GSCs, the expression of SPT6/BRCA1 in DGCs is low, thereby enabling aberrant recruitment of 53BP1 in S/G2 cells and activating error-prone DSB repair via NHEJ (dNHEJ) which associates with increased polyploidy (Fig. 5j, k). Interestingly, polyploidy has been associated with NHEJ as a primary mode of DSBs repair, where Zheng et al.^35^ measured a twenty-fold increase in NHEJ activity in the nuclear extracts from near-polyploid aneuploid cells compared to those prepared from diploid cells. Chromosomal instability and ploidy degree affect the tumorigenicity of GSCs^52^. Our data directly compare the ploidy of matched GSCs and DGCs (Fig. 5k), and imply that the baseline differences in the percentage of polyploid cells may be a consequence of differential DSBs repair pathway choice in these two sub-populations.

The interaction of SPT6 with RNAP II is important for complete recruitment of the SPT6/RNAP II complex across genes and thus for a proper mRNA turnover^23^. SPT6 is essential for the phosphorylation of C-terminal domain of RNAP II (CTD) at Ser2 and that mutation in the CTD results in loss of SPT6 protein stability^24^.

We show that SPT6 loss leads to proteasomal degradation of RNAP II which in turn reduces global transcription rates in general, and the expression of BRCA1 (mRNA and protein), in particular (Fig. 4). These results corroborate previous findings, based on transcriptional profiling of SPT6 silenced HELA cells followed by SPT6-ChIP analysis, that validate BRCA1 as a direct transcriptional target of SPT6^53^. Our study not only validates their observation, but also provides a mechanistic insight into the consequences of SPT6 knockdown-mediated BRCA1 downregulation on genomic stability in the context of cellular hierarchies using unique GBM models. Another study has shown that cell-cycle-associated transcripts are among the most highly stabilized mRNAs in the absence of SPT6-RNAP II interaction, causing severe cell cycle defects in the SPT6_tSH2_mutants^23^. This is concordant with our results, where SPT6 loss led to a G1-S transition defects and cell cycle arrest at G2 phase (Fig. 3b and Supplementary Fig. 3a).

Elongator and Suppressor of Ty4 (SPT4)/SPT5 are transcription elongation factors that contribute to the regulation of mRNA synthesis by RNA polymerase II in the chromatin context. During the process of immunoglobulin class switch recombination (CSR), the interaction of transcription elongation factor SPT5 with activation-induced cytidine deaminase (AID) is crucial for the recruitment of AID to its Ig and non-Ig targets^54^. SPT5 together with SPT4 were implicated in the regulation of both HR and NHEJ^26^. SPT5 loss in zebra fish was associated with G1 cell cycle arrest and transcriptional changes affected genes involved in diverse biological pathways such as stress response and cell fate specification, but not DNA repair^55^. In HELA cells, SPT5 knockdown induced expression changes that impacted RNA metabolism, cell cycle leading to G1 arrest, senescence and subsequently cell death by apoptosis^56^. Even though SPT6 is not the only transcription elongation factor implicated in DNA repair, it is the only one executing its function via transcriptional upregulation of DNA repair genes.

By subjecting the SPT6 data set (Supplementary Data 3 and 7) to CMap analysis, we identified a number of drugs mimicking SPT6 loss phenotype. Number one on the list was idarubicin, as chemotherapeutic agent and an anthracycline antileukemic drug, which interferes with topoisomerase II and has already been clinically tested as monotherapy in GBM^57^. The second-highest scoring drug was a Polo-like Kinase (PLK) inhibitor BT-2536, which has been tested in Phase II clinical trial in other solid tumors^58^. The third candidate, which we chose for further validation, chaetocin, is a fungal mycotoxin known to cause unspecific histone demethylation^59^. Chaetocin treatment of GSCs led to: (i) decline in both SPT6 and BRCA1 protein levels; (ii) induced DSBs; (iii) cell cycle arrest; and (iv) increased apoptosis, thereby mimicking the SPT6 knockdown phenotype. The pre-treatment of GSCs with chaetocin abrogated their tumor-initiating capacity in vivo (Supplementary Fig. 7h) and most importantly, chaetocin treatment of tumor-bearing mice significantly extended their survival (Fig. 7g). These observations, together with the previously reported ability of chaetocin to (i) induce ROS in leukemia cells^60^; (ii) attenuate HIF1-activity and thereby angiogenesis in hepatocellular carcinoma xenograft models^61^; and trigger apoptosis in melanoma cells^62^; make chaetocin an attractive alternative for therapeutic intervention in therapeutically resistant GBM.

In this study, we describe an oncogene-like function for SPT6 in GBM, one of the deadliest of solid cancers. Our data reveal that SPT6 is essential for the maintenance and tumor-initiating potential of GSCs as well as their capacity to repair endogenous and/or irradiation-induced DNA damage. Moreover, our work has unveiled a previously unknown mechanistic insight into the role of SPT6 in DNA repair in the context of cellular hierarchies. The genetic targeting of SPT6 by shRNA and the use of chaetocin as the ‘mimic’ for SPT6 loss in a pre-clinical orthotopic xenograft model impaired the tumor growth in vivo and significantly improved the survival of tumor-bearing mice. In addition, our work describes the regulatory mechanism by which SPT6 dictates the DSB repair pathway choice via transcriptional upregulation of BRCA1, thereby promoting the error-free HR and genomic stability of GSCs (Fig. 7h). Collectively, our study sheds light on the functional implication of SPT6 in tumorigenesis, providing sufficient evidence for its role as a putative therapeutic target in GBM.

## Methods

### Primary GBM lines, xenograft passaging, and commercial lines

All cell models were derived from tissue specimens obtained after surgery in patients diagnosed with GBM. Primary GBM line T4121 (GBM01) was acquired from J.N Rich (University of California, San Diego, CA, USA) Rich lab, in accordance with Ohio State University or Cleveland Clinic Institutional Review Board. GBM02 and GBM03 were established from freshly resected tumor tissue including informed consent from each patient, as outlined by the Regional Danish Ethical Committee/Danish Data Protection Agency (H-3-2009-136_63114). GBM lines were passaged as xenografts in the subcutaneous flank of NOG mice (Taconic, TAC:nog) according to Danish Welfare Law on Animal Experiments Act no 1306, protocol: 2012-15-2934-00636.

All GBM lines were authenticated to be unique by ATCC STR profiling and routinely tested for mycoplasma.

U2OS and HEK293T cells were purchased from ATCC and maintained as adherent cultures in DMEM with 10% FBS. U2OS-BRCA1-GFP cells were a gift from J. Bartek (Danish Cancer Society, Denmark). U2OS DR-GFP and U2OS EJ5-GFP cells were a gift from Dr. Pablo Huertas (University of Seville, Spain)^63^. Normal Human Astrocytes-Hippocampal (NHA33 and NHA59) were purchased from 3H biomedical (SC1830, 3H Biomedical) and maintained at low passages (max passage 7) in commercially available Complete Astrocyte medium (SC1801, 3H Biomedical) according to manufacturer instructions. All cells were cultured at 37 °C in an atmosphere of 95% air and 5% CO_2_.

### In vivo patient-derived GBM studies

All intracranial studies were carried out according to Danish Welfare Law on Animal Experiments Act no 1306, protocol: 2012-15-2934-00636. For in vivo survival studies, a total of 5 × 10^4^ live cells transduced with either SPT6 shRNA or shLuciferase (shLuc) were stereotactically implanted into right frontal lobe of wild type, female Balb/c nu/nu mice (5 mice/group, 8 weeks). For chaetocin (CHAE, Selleckchem S8068) studies: (A) tumor initiation study: 1 × 10^4^ GBM01 GCSs CHAE-pretreated cells (30 nM; 24 h) were stereotactically implanted into right frontal lobe of 6 weeks old female NMRInu/nu mice (seven mice per arm); (B) therapeutic efficacy study: 1 × 10^4^ GBM01 GCSs were stereotactically implanted into the right frontal lobe of 6-week-old female NMRInu/nu mice (five mice per arm) and randomized into two groups. At day 3 post-implantation, mice were intraperitoneally treated with vehicle control (DMSO) or 1 mg kg^−1^ chaetocin three times a week for a period of 2 weeks (total dose of 6 mg kg^−^^1^). All mice were kept in the same conditions, with access to food and water ad libitum in a 12-h light/dark cycle. Mice were monitored and sacrificed by an animal caretaker blinded to study design at the onset of neurological impairment, loss of balance and appetite, hunching, weight loss. Mice that remained asymptomatic were sacrificed at the end of respective study (85 days post-implantation for SPT6 shRNA study in Fig. 2f, and chaetocin treatment study in Fig. 7g; 105 days post-implantation for chaetocin pre-treatment study (Supplementary Fig. 7h).

### Tumor dissociation and GBM cell culture

Freshly resected xenografts were dissected and mechanically dissociated according to the Papain Dissociation protocol (Worthington Biochemical Corporation, New Jersey, cat.no.LK003150). Isolated cells from xenografts were maintained as suspension cultures in complete media containing Neurobasal –A medium (12349-015, Invitrogen), supplemented with B27 minus Vitamin A (12587-010, Invitrogen), EGF (20 ng ml^−1^) (236-EG-01M, R&D systems), FGF (20 ng ml^−1^) (4114-TC-01M, R&D systems), GlutaMax (35050-038, Invitrogen) and antibiotics (15140-122, Invitrogen). Cells were allowed to recover for 24 h prior their use in downstream experiments. Matched GBM cancer-stem like cells (GSCs, CD133+) and differentiated GBM cells (DGCs, CD133−) populations were isolated by magnetic (MACS) sorting using CD133 microbeads kit (130-100-857, Miltenyi Biotec). The GSCs were maintained in complete media, whilst the DGCs were maintained as monolayer in DMEM (31966-021, Invitrogen), supplemented with 10% FBS (medium (15140-122, Invitrogen) and antibiotics. Both populations were validated functionally by sphere-forming capacity (ELDA) and expression of stem cell markers by immunoblotting for expression of SOX2 and GFAP.

### siRNA screen and analysis

A customized siRNA library consisting of 296 gene targets with known role in chromatin remodeling, positive (INCENP9) and negative controls (scrambled siRNA control), with three independent validated siRNAs per gene (Supplementary Data 1) was purchased from Ambion. A pool of three individual siRNAs for each gene, to a final concentration of 900 nM per well, was used to transfect a total of 4 × 10^5^ cells using a 384-well Nucleofector^TM^ System and P3 Nucleofector^TM^ Solution (Lonza). After transfection, cells were transferred to a GelTrex-coated (A1413201 ThermoFisher) 384-well imaging plate (Nunc) in growth factor (EGF, bFGF) free Neurobasal-A medium. 72 h later, cells were pulse-labeled (30 min) with 5-ethynyl uridine (EdU; A10044, Life Technologies), then fixed with 4% PFA, permeabilized with 0.25% triton x-100 in PBS and incubated with anti-γH2AXSer139 antibody for 1 h at RT. The incubation with secondary anti-mouse Alexa Flour 488 conjugated antibody (9669S, Cell Signaling) was followed by EdU detection according to the manufacturer’s instructions (A10044, Life Technologies). Nuclei were counterstained with 4,6-Diamidino-2-Phenylindole (DAPI) (D9542-10MG, Sigma Aldrich). Acquisition and analysis were carried out using Olympus Scan-R screening station equipped with Scan-R analysis software (Olympus).

### RNAi knockdown, plasmid transfection, and treatment

GBM cells were wet-reverse transfected using either HiPerfect Transfection Reagent (301705, Qiagen) or Dharmafect1 (T-2001-03, GE Healthcare) according to manufacturer’s instructions. For each transfection, 2 × 10^5^ cells were transfected with 50 nM of either pooled (siSPT6-p) or individual SPT6 siRNA (Ambion 4392420-Pre-designed siRNA: siRNA-34: S13634; siRNA-35: S13635; siRNA-36: S13636) or 20 nM silencer select negative control siRNA (siCON) (4390844, Thermo Fisher Scientific). For U2OS cells, in a 6 cm dish setting 4 × 10^5^ cells were transfected with siCON (20 nM) or siSPT6 (30 nM) siRNA using Lipofectamine RNAiMax (13778-075, Invitrogen). All analysis and downstream experiments were carried out 72-h post-transfection unless stated otherwise. pEGFP-C1 (Clontech, Cat discontinued) transfection in U2OS cells was carried out using DharmaFECT kb (T-2006-01, Dharmacon) according to manufacturer’s instructions. U2OS-pEGFP-C1-BRCA1 cell line was a gift from Dr. Jiri Bartek (Danish Cancer Society). When indicated, cells we exposed to IR (IR; 3 Gy) using YXLON smart (YXLON International A/S) system and/or treated with indicated doses of chaetocin (stock solution 10 mM) prior processing for downstream assays.

### Lentiviral particle preparation

HEK293T cells were co-transfected with packaging plasmids (pMD2.G and psPAX) and pLKO plasmids (encoding shRNAs targeting SPT6: shSPT6-1: TRCN0000278911, shSPT6-2: TRCN0000278845) or pI-SceI-BFP (gift from Dr. Pablo Huertas, University of Seville, Spain) vector using a Calcium Phosphate transfection kit (631312, Clontech). Lentiviral particles were concentrated using PEG-it Virus Precipitation Solution (SBI, LV810A-1) and stored in aliquots at −80 for later use.

### Clonogenic assay

For colony formation, U2OS cells transfected with either siCON or siSPT6 were plated at a density of 2500 cells per well in a 6-well plate and allowed to attach overnight. The following day, cells were irradiated at 3 Gy or sham-irradiated and monitored for 6 days. For analysis, cells were fixed and stained using 0.5% crystal violet(w/v) (C0775-25G, Sigma Aldrich), scanned. Colonies were quantified using Celigo Imaging Cytometer (Nexcelom Bioscience).

### Flow cytometry

Apoptosis: In brief, single cells were incubated with Annexin V-FITC (Dead cell apoptosis kit; V13242, BD bioscience) and processed according to the manufacturer’s instructions. For Cleaved Caspase 3 staining, single cells were fixed in 4% PFA, permeabilized, stained at 37 °C for 90 min with antibody against cleaved caspase 3 (Asp175) conjugated with Alexa Flour 488 (9669S, Cell Signaling). Cell nuclei were counterstained with DAPI.

Polyploidy: Here, single cells were fixed in 4% PFA for 10 min then permeabilized in 0.25% triton-X in PBS for 10 min. The DNA content was visualized by incubating the fixed cells with DAPI staining solution (10 μg ml^−1^ in 0.1% triton-X in PBS) for 2 h before analysis.

Proliferative index or S phase (EdU^+^ cells): Cells were pulse-labeled with 10 µM EdU probe for 30 min, fixed using 4% PFA for 15 min and stained using Click-iT EdU imaging kit according to manufacturer’s instructions (A10044, Life Technologies). Nuclei were counterstained using DAPI.

Mitotic index (MI): Single cells were collected, fixed with 4% PFA, then incubated with phospho-histone H3 (Ser10) Alexa Flour 647 conjugated antibody (Cell Signaling Cat#9716). Cell nuclei were counterstained with DAPI.

Global transcription rates: Cells were pulse-labeled with EU probe (1 mM) for 1 h and stained using the Click-it RNA Alexa Flour 488 kit (C10329, Life Technologies) according to manufacturer’s instructions.

HR/NHEJ repair rates: Upon transfection with HR or NHEJ repair plasmids, 48 h later cells were trypsinized and single-cell population analyzed for the frequency of GFP positive cells.

All flow cytometry samples were run on BD FACS Verse (BD Biosciences) and analyzed using FlowJo software (BD). Information on gating strategy is provided in Supplementary Fig. 9.

### Immunofluorescence

For immunofluorescence staining, cells grown on GelTrex-coated coverslips were fixed with 4% PFA, permeabilized using 0.25% Triton-X or pre-extracted for 3 min using a mix of 4% PFA and CSK buffer (20 mM NaCl, 5 mM MgCl_2_, 1 mM PIPES, 1 mM EGTA, 100 mM Sucrose and 0.2% Triton-X) in ratio 1:1, prior fixation with 4% PFA, followed by blocking in 3% BSA. Cells were incubated with primary antibody: rabbit polyclonal anti-Cyclin A (H-432) (sc-751, Santa Cruz, mouse monoclonal anti BRCA1(D-9) (sc6954, Santa Cruz), Rabbit polyclonal anti-53BP1 (ab36823, Abcam), mouse monoclonal anti-H2AXSer139 (05-636, Millipore) or rabbit polyclonal anti SPT6 (Ab32820, Abcam), at 4 °C overnight or 2 h at RT, followed by incubation with appropriate AlexaFlour488 or AlexaFlour568 conjugated secondary antibody (1:1000). Nuclei were counterstained with 4,6-Diamidino-2-Phenylindole (DAPI) (D9542-10MG, Sigma Aldrich).

For the two-dimensional analyses of 53BP1 and BRCA1 foci counts in the context of cell cycle phases, cells were either pulse-labeled with EdU followed by staining with a Click-iT EdU imaging kit (C10337, Life Technologies) or just stained with antibody against cyclin A (to mark S/G2 phase cells)^9^. Automated imaging and analysis were performed using Olympus Scan-R screening station equipped with Scan-R analysis software. Where matched GSCs and DGCs were assessed. To correct for differential nuclear area/size in matched GSCs and DGCs, the absolute foci count per cell nucleus was normalized to Nuclear Area (NA). Confocal images were acquired using Zeiss LSM800 confocal microscope.

### Cell viability and GI_50_ calculation

For cell viability was cells transfected with siCON, siSPT6-p or individual siRNAs (siSPT6-34, siSPT6-35 or siSPT6-36) were seeded at a density of 3000 cells per well in 100 μl of media in 96-well plates, all in triplicates. Cell viability was measured at indicated time points using CellTiter-Glo Luminescent Cell Viability Assay (G7571, Promega). For GI_50_ studies, single cells were seeded at 3000 cells per well in 50 μl of media in 96-well plates. After 24 h, chaetocin (increasing concentrations as indicated) was added to a final volume of 100 µl. After 72 h, viability was measured using CellTiter-Glo Luminescent Cell Viability Assay (G7571, Promega).

### Extreme limiting dilution assay (ELDA)

Self-renewal of GSCs was assessed by ELDA^64^. In brief, cells transfected with siCON, siSPT6-p or individual siRNAs (siSPT6-34, siSPT6-35 or siSPT6-36) were seeded in 96 well plate at a density of 500, 250, 100, 50, 10, and 1 cells per well. After 14 days, the presence of neurospheres in each well was noted and data analyzed using the ELDA software (http://bioinf.wehi.edu.au/software/elda).

### Immunoblot analysis

Protein extracts were prepared using whole lysis buffer (WLB; 50 mM Tris –HCl, 10% Glycerol, 2% SDS and water) and protein concentrations determined by Pierce BCA Protein Assay (23227, Thermo Scientific). Cell lysates (25–30 µg) were separated by electrophoresis on SDS-PAGE gels (BioRad). Proteins were transferred onto nitrocellulose membranes and incubated appropriate primary and species-specific secondary antibodies (see Supplementary Table 1). Amersham ECL Prime Western Blotting Detection Reagent (RPN2232, GE Healthcare) was used for detection using Image Lab software (BioRad). Uncropped immunoblot images are provided in Supplementary Fig. 8.

### Alkaline comet assay

Single cells were resuspended in 0.5% low melting agarose (LMA) (A9045-10G, Sigma), spread quickly onto 1% Ultra-Pure normal melting agarose (NMA) (16500500, Invitrogen) pre-coated glass slides. Slides were immersed into lysis buffer (2.5 M NaCl, 100 mM EDTA and 10 mM Trizma base) overnight at 4 °C. Slides were then washed in neutralization buffer (Trizma base, PH = 7.5, calibrated with HCl) to quench lysis and run in an electrophoresis chamber at 25 V, 300 mA for 25 mins in a cold room, dehydrated in 96% ethanol, and mounted in TE buffer with SYBR Gold (S11494, Thermo Fisher Scientific). Comets were imaged by a fluorescence microscope (Axiovert 200 M, Carl Zeiss) and comet tails scored using with Comet assay IV software.

### Immunohistochemistry

Paraffin-embedded, neoplastic, tumor tissue (8 µm; *n* = 24) and non-malignant control brain (8 µm; *n* = 10) sections were obtained from the Department of Clinical and Molecular Pathology, Palacky University and University Hospital Olomouc upon acquisition of a valid consent per the requirement of regional ethics committee and stained as described previously^9^. In brief, following antigen retrieval, tissue sections were treated in 6% H_2_O_2_ to block endogenous peroxidase activity, then stained with primary antibody against SPT6 (1:100; ab32820, Abcam). After 1-h incubation at room temperature (RT), sections were washed, incubated with EnVision+ Dual Link System-HRP secondary antibody for 1 h, and immunoreactivity visualized using liquid DAB + substrate-chromogen system. Slides were washed, dehydrated through graded ethanol and mounted. The nuclei were counterstained with hematoxylin. SPT6 positivity was scored as negative (0), low (1), medium (2), and high (3).

### DSB repair assays

Repair pathways (HR and NHEJ) were assessed using U2OS cells bearing a single copy of the reporter constructs EJ5-GFP and DR-GFP (gifts from Dr. Pablo Huertas, University of Seville) as described by^63^ with minor modifications. U2OS cells were wet-reverse transfected with indicated siRNAs: SPT-6 pool, BRCA1 (Thermo Fisher Silencer Select Pre-designed and validated siRNA S458) and 53BP1 (Sequence Sense 5’->3’ GAU ACU UGG UCU UAC UGG UUU TT) at 30 nM. After 24 h, single cells were seeded at 70% confluency on coverslips and allowed to attach overnight and transduced with I-SceI-BFP lentiviral particles. The following day, cells were washed and fixed 24 h later with 4% PFA and cells double-positive for GFP and BFP were counted.

Repair pathways (HR and NHEJ) in matched GSCs and DGCs were performed according to Lim et al.^65^. Cells (5 × 10^5^) were transiently transfected with respective DNA repair substrate plasmids (linearized *HindIII* pEGFP-N3 for NHEJ and DR-GFP for HR) using DharmaFECT kb DNA transfection reagent. The DR-GFP plasmid was co-transfected with a (1:2) I-Sce1 meganuclease expression plasmid (pCMV-I-SceI). Transfection efficiencies were determined by co-transfection with a circular pEGFP-N3 vector. Cells were analyzed 48 h post transfection by flow cytometry (Becton Dickinson).

### RNA extraction and real-time PCR

Total RNA was extracted using and RNeasy Plus Mini Kit (QIAGEN-74134) according to the manufacturer’s instructions. cDNA was synthesized from total RNA using the High Capacity cDNA reverse transcription kit (Applied Biosystems). Real-time PCR was performed using FAST SYBR Green Master mix (Applied Biosystems) according to the manufacturer’s instructions. Amplification was performed in the Applied Biosystems 7500 Fast Real-Time PCR System. Primer sequences are listed in Supplementary Table 2. HPRT1 was used as house-keeping control and ΔΔCT method was used to calculate fold change expression^9^.

### ChIP qPCR

For chromatin immunoprecipitation 5 × 10^6^ GSCs were plated and recovered overnight, then crosslinked in 1% PFA in cell culture media for 10 mins with gentle shaking. Glycine (125 mM) was added to mixture for 10 min at room temperature (RT). Next, cells were washed twice in ice-cold PBS cold and pelleted at 1400 rpm for 5 min. prior lysis^43^. The isolated chromatin fraction was fragmentized using the Covaris M220 (27 min; peak power: 75; duty factor: 10; cycles/burst: 200). Soluble chromatin fraction was isolated by centrifugation at 13,000 rpm at 4 °C for 10 min. For immunoprecipitation, Protein Dynabeads G were washed and incubated for 6 h with IgG, at 4 °C on a rotating wheel. The chromatin was precleared by incubation with a mixture of beads and IgG for 30 mins at RT in rotation. 10% of the precleared chromatin was taken as input control. The pre-cleared chromatin was diluted in dilution buffer (10 mM Tris-HCl pH8.0, 5 mM EDTA, 0.5% Triton X-100 and 0.15 M NaCl) and incubated with ChIP-grade antibody targeting either IgG control- or SPT6 (NB100-2582, Novus Biologicals)—coupled beads overnight at 4 °C on a rotating wheel. Washing was performed using 1 mL of buffer A (20 mM Tris-HCl pH 8.0, 2 mM EDTA, 0.05% SDS, 1% Triton X-100 and 0.165 M NaCl) followed by a wash with 1 mL of buffer B (20 mM Tris-HCl pH8.0, 2 mM EDTA, 0.05% SDS, 1% Triton X-100 and 0.5 M NaCl), 1 mL of buffer C (10 mM Tris-HCl pH8.0, 1 mM EDTA, 1% NP-40, 1% Sodium Deoxycholate and 0.25 M LiCl) and then twice in 1 mL of buffer D (10 mM Tris-HCl pH8.0 and 1 mM EDTA). All washes were performed at 4 °C. Immunoprecipitated chromatin and total input control were decross-linked using 0.01 mg mL^−1^ RNase A in 300 mL of buffer E (1% SDS, 0.1 M NaHCO3 and 0.5 M NaCl) at 65 °C for at least 4 h which gentle shaking at 750 rpm. Next, 30 μL of 10× Proteinase K buffer (200 mM Tris-HCl pH 6.5, 150 mM EDTA and Proteinase K 0.3 mg mL^−1^) was added and samples were then incubated 45 °C for 2 hrs. The DNA fragments were purified using phenol/chloroform (pH 7.0) and ethanol precipitation. Eluted DNA was used as a template for qPCR analysis using Applied Biosystems 7500 Fast Real-Time PCR Systems. BRCA1 primers (Supplementary Table 2) were designed based on the human BRCA1 distal promoter.

### In silico analyses of public data sets

The pre-processed CPM normalized data from Suva data set^28^ was downloaded from GEO (GSE54791). The CPM matrix was log2 transformed with a pseudo-count of 1 and re-analyzed for differential gene expression (GSCs vs DGCs, called ‘TPC’ and ‘DGC’ by Suva et al.) using a standard limma DE workflow with minor modification to account for the non-independence of the replicates from the same patient-derived model^66^. The black trend line is calculated as a smoothed average along the x-axis where the shaded region indicates standard error. Results are summarized in Supplementary Data 5. The differential SPT6 mRNA expression in NB in comparison to malignant gliomas and its impact on patient survival was assessed using indicated data sets via http://gliovis.bioinfo.cnio.es/^67^.

### RNA sequencing and analysis

Total RNA isolated from GBM01 GSCs transfected with either siCON or siSPT6-p using RNeasy Plus Mini kit (QIAGEN #74134) was subjected to a library preparation and pair-end sequencing using BGI services (https://www.bgi.com).

Trimming and mapping: The following analysis service was provided by GATC Biotech: Reads were timed using Trimmomatic v0.33 and were mapped to hg19 with TopHat 2.0.14 guided by Gencode v19.

Quantification and filtering: Gencode v19 Gene expression was quantified via featureCount only counting reads mapping uniquely to individual gene. Inter library normalization was done using with edgeR 3.14.0 using the ‘TMM’ method. Only genes expressed more than 1 RPKM in at least three samples were kept for subsequent analysis (*n* = 13,693).

Differential gene expression: Genes were tested for differential expression using edgeR’s glmLRT function with a design accounting for the paired nature of the independent experiments (Supplementary Data 3). *P*-values were corrected for multiple testing using the Benjamini-Hochberg approach. An FDR value < 0.05 was considered significant.

Gene set overrepresentation analysis (OA): Gene set annotation was obtained from Gene Ontology^68^ and MSigDB v5 (http://bioinf.wehi.edu.au/software/MSigDB/) and only the sets H (Hallmark sets), C2 (Curated sets) and C6 (Oncogenic signature sets) were used. Gene Ontology gene set were downloaded from EBI’s official mirror January 2016 and to avoid the bulk of gene-duplication only gene sets at level 6 of the biological process hierarchical ontology structure was used (referred to as the GO gene set). Ensemble IDs were translated to gene-names using biomaRt. Overrepresentation was done via R’s fishers.exact() test with alternative = greater (Supplementary Data 4). *P*-values were corrected for multiple testing using the Benjamini-Hochberg approach (FDR). An FDR value < 0.05 was considered significant.

Clustering of gene set overrepresentation: Up- and downregulated results were clustered separately and only gene sets significantly overrepresented (FDR < 0.01) was used for the clustering. To cluster gene sets a graph connecting all gene sets to all gene sets was constructed. The score of each connection was measured as the Jaccard similarity (the fraction of genes in the union of the two gene sets which were found in both). The graph was trimmed by only keeping connections with a Jaccard similarity > = 0.15 and with at least 5 genes in the overlap. The Louvain method (implemented in the igraph R package) was used to identify clusters of overlapping gene sets. We derived the title of each cluster via the natural language processing tools implemented in the R package tidytext. For each gene set we separated the gene set name into its distinct words. The words: reactome, kegg, dn, up, network, corr; were added to the list of common stop-words (which were removed) and the text mining statistics tf-idf was calculated for each word in each cluster. In the context of gene set clusters the tf-idf statistics is a convenient way of identifying the words which best described one gene set cluster compared to the words of gene sets from all other clusters, while at the same time taking the word frequency into account (for details see https://www.tidytextmining.com/tfidf.html). For each cluster we extracted the top 10 words with the highest tf-idf score and from those the cluster titles were manually constructed. Only clusters containing gene sets from more than one source were annotated (else marked NA). To improve the visual presentation 2 “unclear” unconnected clusters, as well as the following 3 unconnected clusters, were removed from the visualization: Aminoacytelation, Monosaccharide processes and ERBB2 and MEK signaling. The entire process is documented in Supplementary Data 6.

Analysis of specific gene sets: DNA repair gene sets were extracted by selecting all gene sets containing one of the following words: repair, homologous, nonhomologous or non_homologous. Stem-cell gene sets were extracted by selecting all gene sets containing stem_cell in its name and afterwards remove all sets containing either of the following (sub)words in the description: hematopo, mammary, leukemic or lymphoid. Afterwards the top 250 upregulated genes (ranked by *p*-value) from the Suva data set was added as a stem-cell gene set.

### Connectivity Map (CMap) analysis

We used The Connectivity Map (CMap)^38^ to identify compounds mimicking transcriptional perturbation caused by SPT6 knockdown (Supplementary Data 7). Specifically, we extracted the top 500 most up- and downregulated genes (sorted by *p*-value) after SPT6 silencing, converted the gene_IDs to Entrez_IDs and uploaded them to the next generation CMap server hosted at https://clue.io/.

### Statistical analysis

The choice of sample size is similar to previously reported studies^9,11,69^. GraphPad Prism 8 was used to assess statistical significance in every case. For in vivo studies, Kaplan–Meier survival curves, log-rank test was performed. For in vitro studies, statistical significance was assessed by two-tailed unpaired Student’s *t*-test, or by one-way or two-way ANOVA followed by a post-hoc analysis as indicated in the figure legends. For correlation analyses Spearman’s rank correlation test was performed. Precise details on number of independent experiments or samples and statistical tests can be found in the figure legends. *p* values < 0.05 were considered statistically significant.

### Reporting summary

Further information on research design is available in the Nature Research Reporting Summary linked to this article.

## Supplementary information

Supplementary Information

Description of Additional Supplementary Files

Supplementary Data 1

Supplementary Data 2

Supplementary Data 3

Supplementary Data 4

Supplementary Data 5

Supplementary Data 6

Supplementary Data 7

Reporting Summary

## Data Availability

The raw FASTQ files, as well as the gene count matrix have been submitted to NCBI’s Gene Expression Omnibus (GEO) and have been assigned the accession number GSE125621 and permanent URL: https://www.ncbi.nlm.nih.gov/geo/query/acc.cgi?acc=GSE125621. The primary GBM cell lines can be shared with other investigators via fully executed Material Transfer Agreement and Data Transfer Agreement approved by the Danish Data Protection Agency. Uncropped images of individual immunoblot membranes and FACS gating strategies are provided as a Supplementary Figs. 8 and 9. Source Data are provided with this paper and contains key raw data presented in Figs. 1–7 and in Supplementary Figs. 1–7. Antibodies and primers used in the study are listed in Supplementary Tables 1 and  2. The data that support the findings in this study are available within the Article, Supplementary Information or from the corresponding author upon reasonable request. Source data are provided with this paper.

## Source data

Source Data
