# Supplementary material for: SPT6-driven error-free DNA repair safeguards genomic stability of glioblastoma cancer stem-like cells
Source: Nat Commun. 2020 Sep 18;11:4709. doi: 10.1038/s41467-020-18549-8 (PMC7501306; doi:10.1038/s41467-020-18549-8)
Supplement: Supplementary file 1 — Supplementary Information [file 41467_2020_18549_MOESM1_ESM.pdf]

Supplementary Information

SPT6-driven error-free DNA repair safeguards genomic stability of glioblastoma cancer stem-like cells

Obara et al.

Supplementary Figure 1

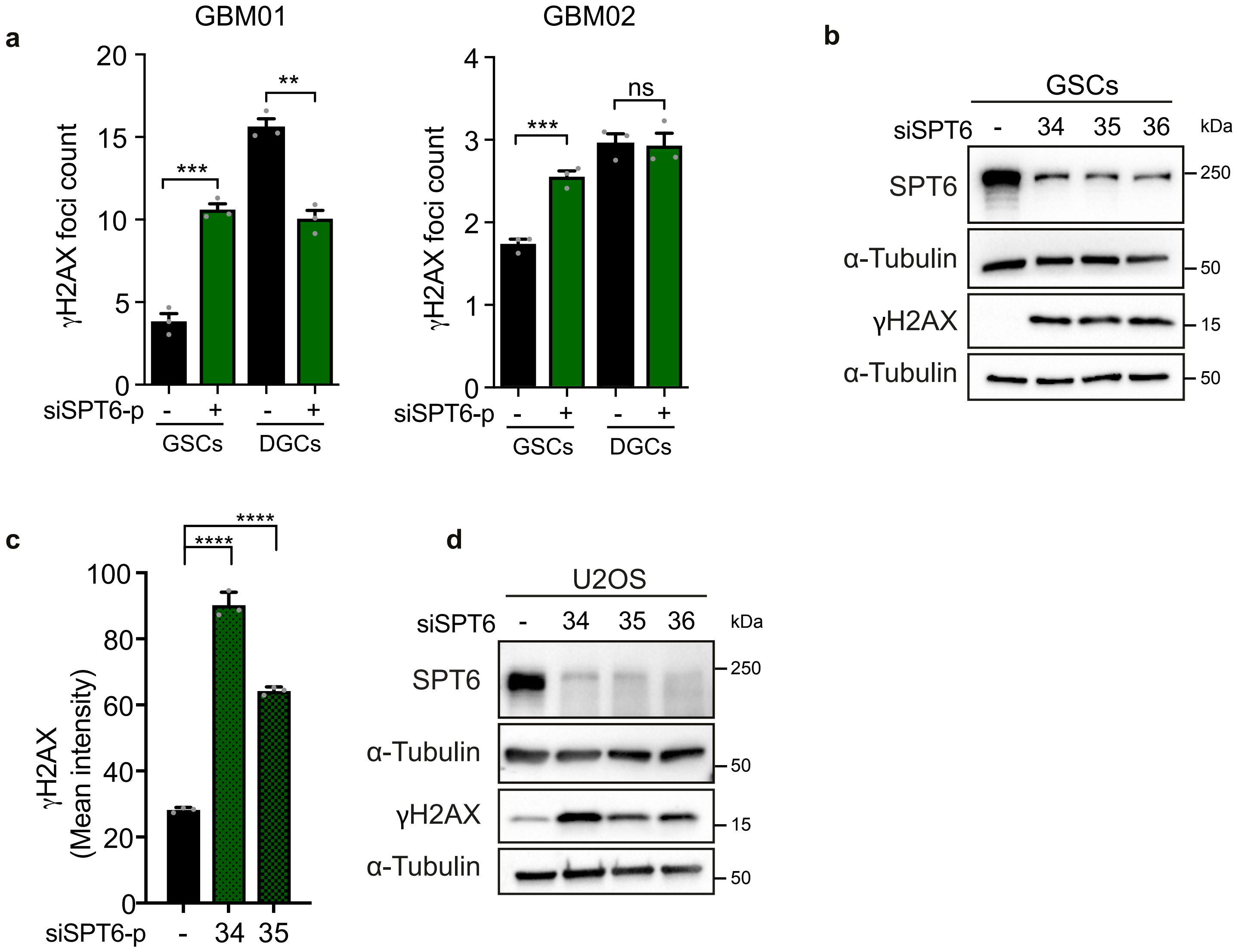

Supplementary Fig. 1: Figure supplementary to main Figure 1

(**a**) γH2AX foci quantification in matched GSCs and DGCs (GBM01) transfected with siCON or siSPT6-p. Data are presented as mean values ± s.e.m. ****p*<0.0003 (GBM01-GSCs), ***p*<0.0013 (GM01-DGCs), ****p*=0.0009 (GBM02-GSCs), non-significant p=0.853 (GM02-DGCs); two-tailed unpaired t-test.

(**b**) Representative immunoblot analysis of SPT6 and γH2AX in GSCs (GBM01) transfected with siCON or three individual siRNAs targeting SPT6 (siSPT6-34, -35 and -36).

(**c**) γH2AX mean intensity quantification in GSCs transfected with siCON or two individual siRNAs targeting SPT6 (siSPT6-34, -35). Data are presented as mean values ± s.d. **** *p*<0.0001; one-way ANOVA analysis followed by Dunnett's multiple test.

(**d**) Representative immunoblot analysis of SPT6 and γH2AX in U2OS cells transfected with siCON or three individual siRNAs targeting SPT6 (siSPT6-34, -35 and -36).

N=3 biological independent experiments in a)-d). Loading control: α-Tubulin in b) and d). Source data are provided as a Source data file.

Supplementary Figure 2

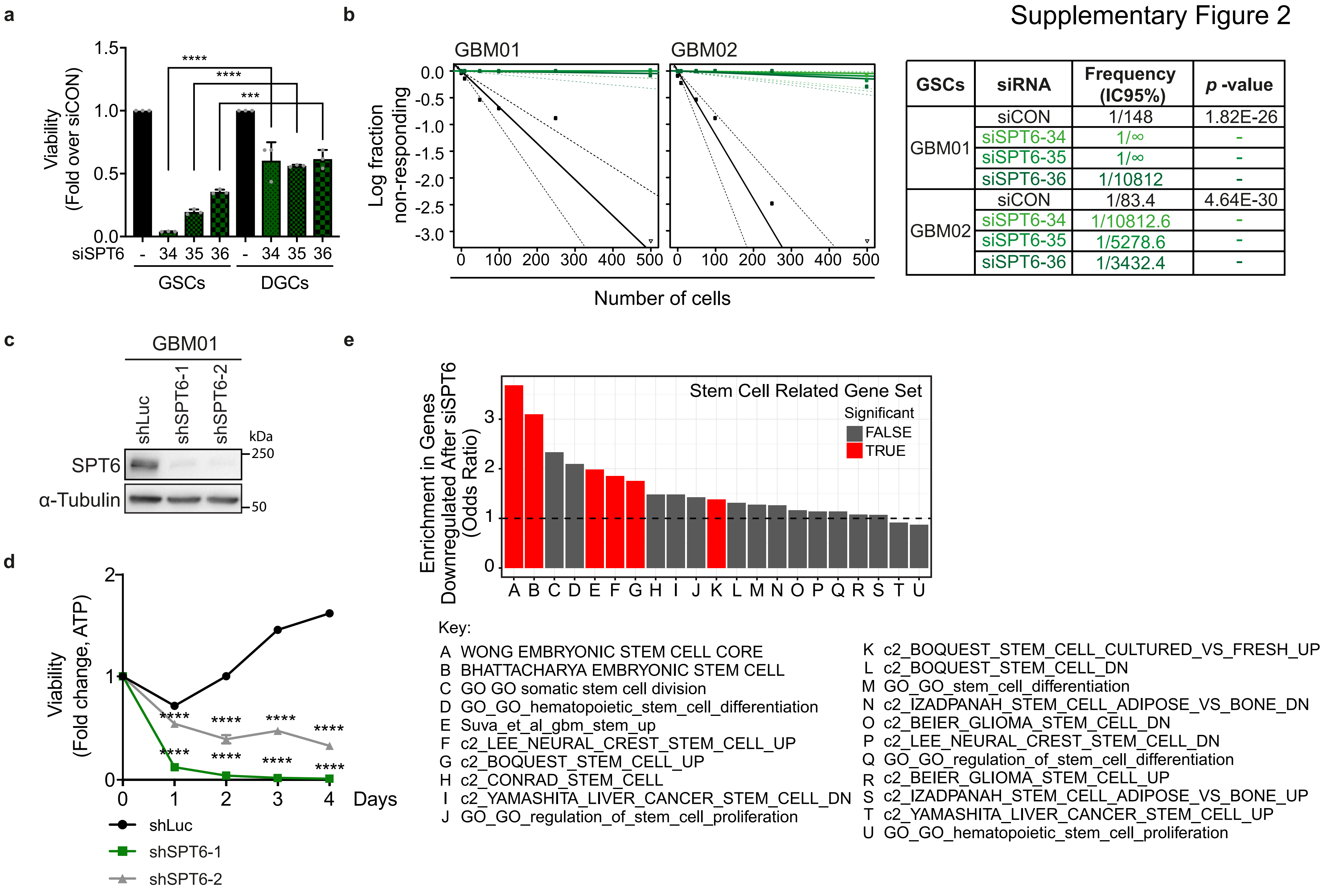

GSCs	siRNA	Frequency (IC95%)	*p* -value
GBM01	siCON	1/148	1.82E-26
	siSPT6-34	1/∞	-
	siSPT6-35	1/∞	-
	siSPT6-36	1/10812	-
GBM02	siCON	1/83.4	4.64E-30
	siSPT6-34	1/10812.6	-
	siSPT6-35	1/5278.6	-
	siSPT6-36	1/3432.4	-

Supplementary Fig. 2: Figure providing supplementary information to main Figure 2

(**a**) Viability assay of matched GSCs and DGCs (GBM01) transfected with either siCON or three independent siRNAs (siSPT6-34, -35 and -36) at day 3 post-transfection. Data are presented as mean values ± s.d. ****p<0.0001, ***p=0.002; two-way ANOVA analysis followed by Sidak's multiple comparisons test.

(**b**) Representative Extreme Limiting Dilution Assay (ELDA) of GSCs (GBM01 and GBM02) transfected with transfected with either siCON or three independent siRNAs (siSPT6-34, -35 and -36). Graphs are accompanied by a table summarizing neurosphere formation frequency and respective *p*-values (GBM01 p=1.82e-26 and GBM02 p=4.64e-30 by ELDA analysis program).

(**c**) Representative immunoblot analysis of SPT6 expression in GSCs transduced with either shLuc or two independent shRNAs targeting SPT6 (shSPT6-1 and shSPT6-2). Loading control: α-Tubulin.

(**d**) Viability assay of GSCs transduced with either shLuc or two independent shRNAs targeting SPT6 (shSPT6-1 and shSPT6-2) over a period of 4 days. Viability at each time point was calculated as the fold change of each time point over day 0. Data are presented as mean ± s.d. ****p<0.001; two-way ANOVA analysis followed by Tukey's multiple comparison test.

(**e**) Enrichment (odds ratio, y-axis) of stem cell related gene sets (n=21, x-axis) among the genes significantly downregulated after SPT6 (siSPT6-p) silencing(x-axis). The dashed line indicates cut-off for enrichment. Red color indicates significance with *p*-value < 0.05; one-sided Fisher's exact test.

N=3 biological independent experiments in a)-d). Source data are provided as a Source data file.

Supplementary Figure 3

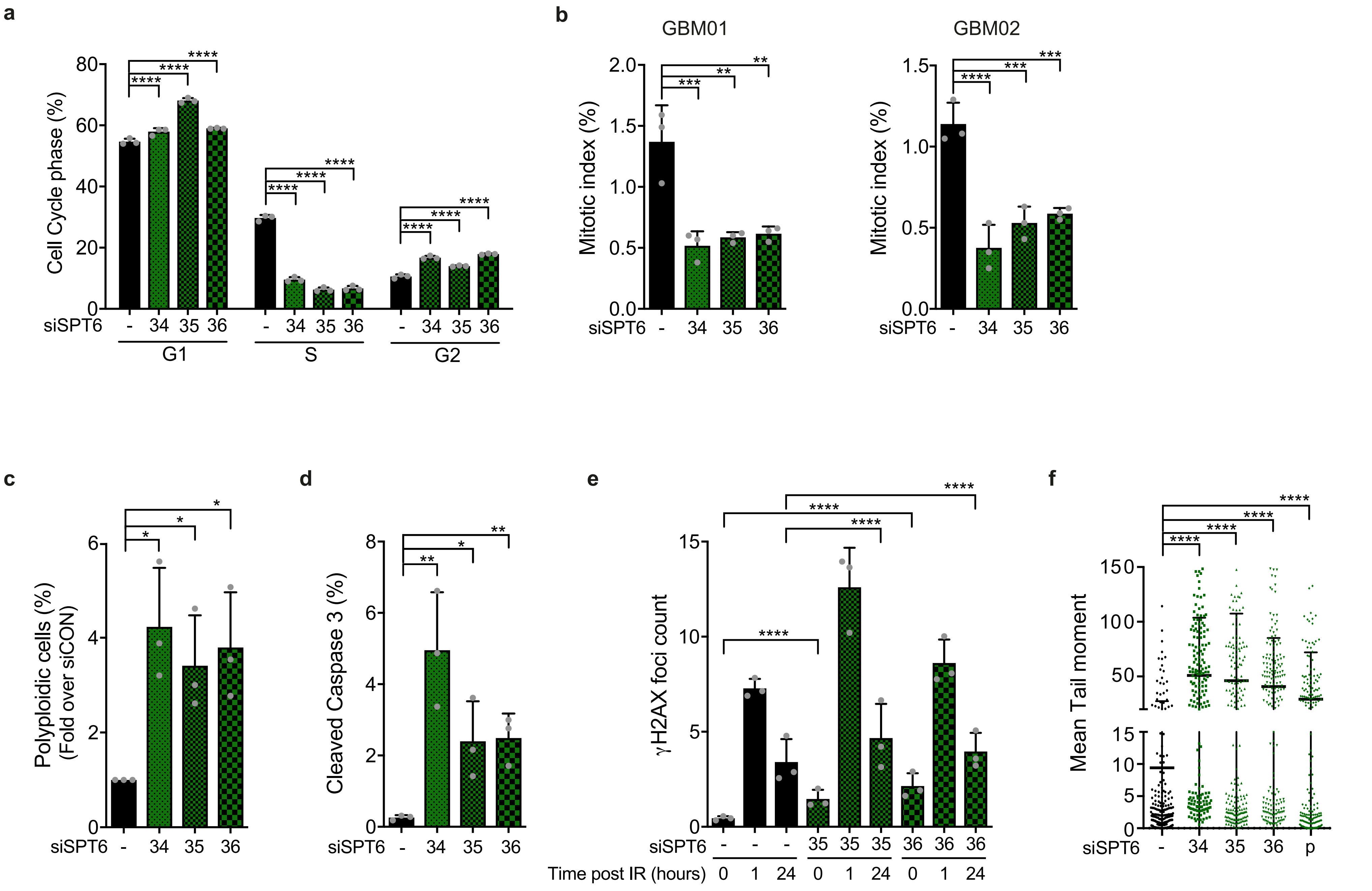

Supplementary Fig. 3: Figure providing supplementary information to main Figure 3

(**a**) FACS analysis of cell cycle profile in GSCs (GBM01) transfected with siCON or three independent siRNAs (siSPT6-34, -35 and -36). Bar graph indicates the % of cells in G1, S and G2. Data are presented as mean values ± s.d. ****p<0.0001; two-way ANOVA analysis followed by Dunnett's multiple comparisons test.

(**b**) Mitotic index (MI) in GSCs (GBM01) transfected with either siCON or three independent siRNAs (siSPT6-34, -35 and -36). Data are presented as mean values ± s.d. GBM01: ***p=0.0006, **p=0.0010, **p=0.0013 and GBM02: ****p<0.0001, ***p=0.0004, ***p=0.0007; one-way ANOVA analysis followed by Dunnett's multiple comparisons test.

(**c**) FACS analysis of polyploid cells fraction (%) in GSCs (GBM01) transfected with either siCON or three independent siRNAs (siSPT6-34, -35 and -36). Data are presented as mean values ± s.d. *p=0.0108, *p=0.0455, *p=0.0230; one-way ANOVA analysis followed by Dunnett's multiple comparisons test.

(**d**) FACS analysis of cleaved caspase-3 in GSCs (GBM01) transfected with either siCON or three independent siRNAs (siSPT6-34, -35 and -36). Data are presented as mean values ± s.d. **p=0.0075, *p=0.0297, **p=0.0050; two-tailed unpaired t-test.

(**e**) γH2AX foci quantification at 0, 1 and 24 hours after the exposure to ionizing radiation (IR, 3Gy) in GSCs (GBM01) transfected with either siCON or two independent siRNAs (siSPT6-35 and -36). Data are presented as mean values ± s.d. ****p<0.001; two-way ANOVA analysis followed by Tukey's multiple comparisons test.

(**f**) Representative DSBs quantification using a comet assay in GSCs transfected with siCON, siSPT6-p or three independent siRNAs (siSPT6-34, -35 and -36). ****p<0.0001; one-way ANOVA analysis followed by Dunnett's multiple comparison test.

N=3 biological independent experiments in a)-f). Source data are provided as a Source data file.

Supplementary Fig 4

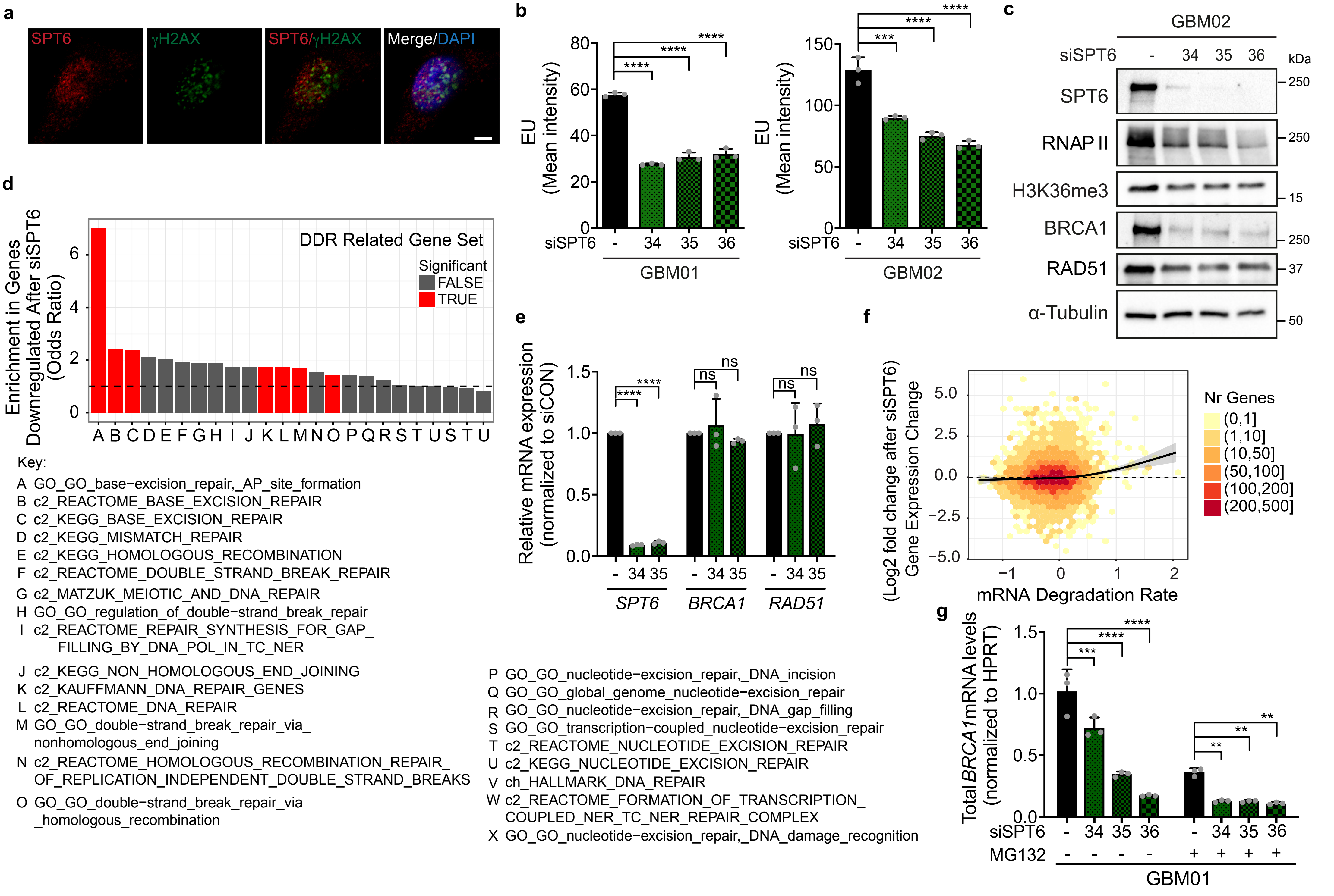

Supplementary Fig. 4: Figure providing supplementary information to main Figure 4

(**a**) Representative confocal microscopy images of immunofluorescence staining of GSCs (GBM01) cells for SPT6 (red) and γH2AX (green). Counterstained with DAPI (blue). Scale bar = 5μm.

(**b**) FACS analysis of global transcription rates in GSCs (GBM01 and GBM02) transfected with either siCON or three independent siRNAs (siSPT6-34, -35 and -36). Data are presented as mean ± s.d. ****p<0.0001 (GBM01), ***p=0.001 and ****p<0.0001 (GBM02); one-way ANOVA analysis followed by Dunnett's multiple comparison test.

(**c**) Representative immunoblot analysis of SPT6, RNAP II, H3K36me3, BRCA1 and RAD51 protein levels in GSCs (GBM02) transfected with siCON or three independent siRNAs (siSPT6-34, -35 and -36). Loading control: α-Tubulin.

(**d**) Enrichment (odds ratio, y-axis) of DNA Damage Repair related gene sets (n=29, x-axis) among the genes significantly downregulated after siSPT6 (x-axis). The dashed line indicates cut-off for enrichment. Red color indicates significance with P-value < 0.05; one-sided Fisher's exact test.

(**e**) qRT-PCR analysis of *SPT6*, *BRCA1* and *RAD51* mRNA expression in DGCs (GBM01) transfected with either siCON or two independent siRNAs (siSPT6-34, -35). HPRT was used as house-keeping gene control. Data are normalized to siCON and presented as mean ± s.d. ****p<0.001 (*SPT6*), non-significant ns=0.7637 and ns=0.7502 (*BRCA1*), non-significant ns=0.9957 and ns=0.6974 (*RAD51*); two-way ANOVA analysis followed by Dunnett's multiple comparison test.

(**f**) Correlation analysis validating that transcriptional changes associated with SPT6 loss are not related to previously-reported half-life of RNAP II-transcribed mRNAs. No significant correlation.

(**g**) qRT-PCR analysis of *BRCA1* mRNA expression in GSCs (GBM01) transfected with either siCON or three independent siRNAs (siSPT6-34, -35 and -36) followed by treatment with DMSO or MG132 (5 hours, 20 μM). House-keeping control gene: HPRT. Data are presented as mean ± s.d. DMSO: ***p=0.00023, ****p<0.0001 and MG132: **p=0.0025, **p=0.0024, **p=0.0013; two-way ANOVA analysis followed by Dunnett's multiple comparison test. N=3 biological independent experiments in a)-c), e) and g). Source data are provided as a Source data file.

Supplementary Figure 5

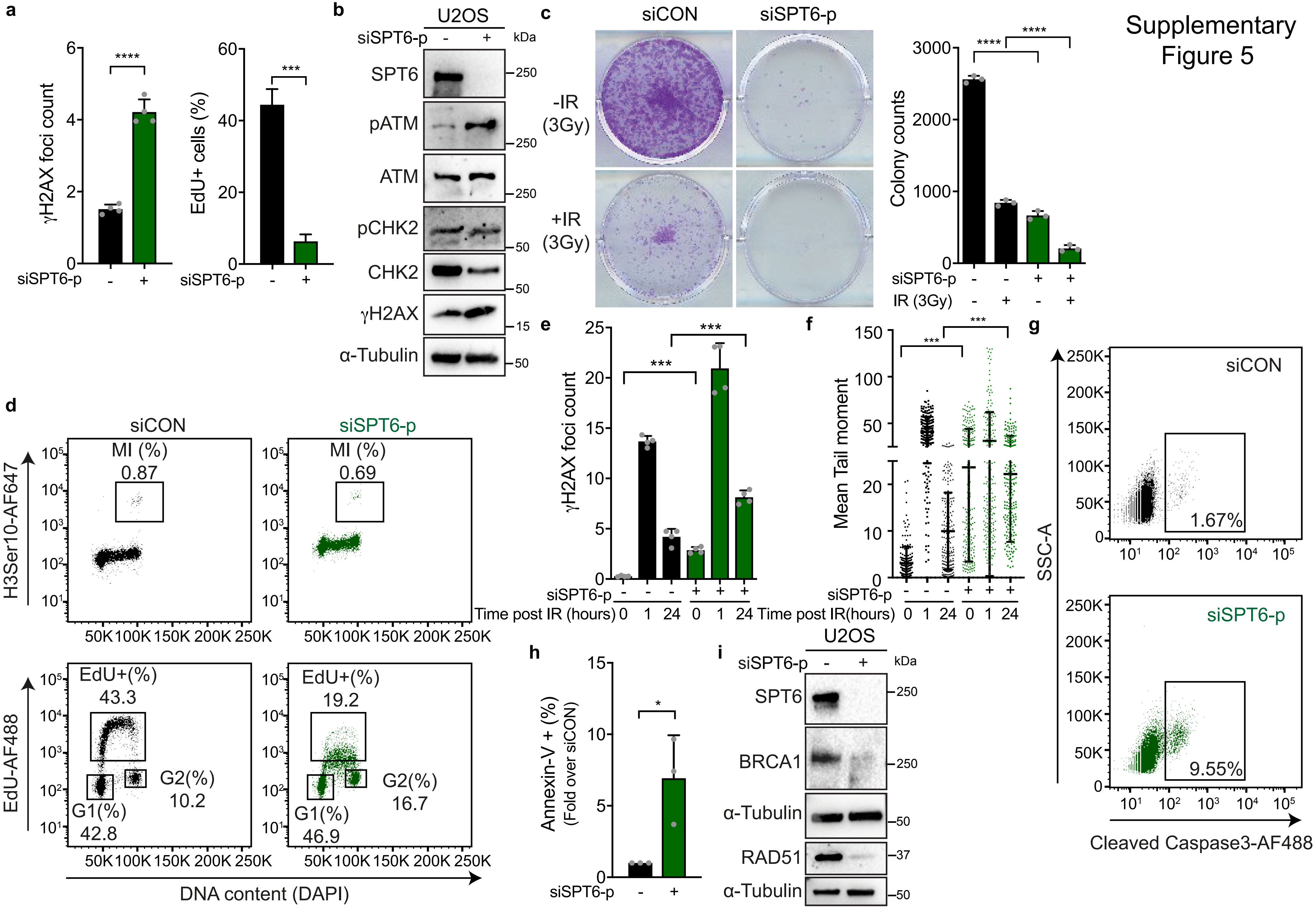

Supplementary Fig. 5: Figure providing supplementary information to main Figure 5a-d

(**a**) Quantification γH2AX foci count and the % of EdU-positive cells in U2OS cells transfected with siCON or siSPT6-p. Data are presented as mean ± s.d. ****p<0.001 and ***p=0.0002; two-tailed unpaired t-test.

(**b**) Representative immunoblot analysis of total and phosphorylated ATM, CHK2, and γH2AX in U2OS cells transfected with siCON or siSPT6-p.

(**c**) Colony formation assay of U2OS cells transfected with siCON or siSPT6-p and exposed to ionizing radiation (3Gy) or sham-irradiated. Graph data are shown as mean ± s.d. ****p<0.001; two-tailed unpaired t-test.

(**d**) Representative FACS plots showing the % of H3Ser10$_+$ cells (Mitotic index; MI), proliferative index (EdU$_+$ cells) and cell cycle distribution of U2OS cells transfected with siCON or siSPT6-p.

(**e**) Microscopy-based quantification of γH2AX foci count at 0, 1 and 24 hours after SPT6 knockdown followed by irradiation (3Gy). Data are presented as mean ± s.d. ****p<0.0001 and ***p=0.0003 (e), ****p<0.0001 (d), two-tailed unpaired t-test.

(**f**) Representative DSBs quantification using comet assay at 0, 1 and 24 hours after SPT6 knockdown followed by irradiation (3Gy). Data are presented as mean ± s.d. ****p<0.0001 and ***p=0.0003 (e), ****p<0.0001 (d), two-tailed unpaired t-test.

(**g**) Representative FACS plots showing the % of cells positive for cleaved caspase-3 in U2OS cells with silenced SPT6 (siSPT6-p).

(**h**) Quantification of Annexin-V-positive U2OS (%) after SPT6 knockdown (siSPT6-p). Data and presented as mean ± s.d. *p=0.027; two-tailed unpaired t-test.

(**i**) Representative immunoblotting analysis of SPT6, BRCA1 and RAD51 protein expression in U2OS cells transfected with siCON or siSPT6-p.

N=3 biological independent experiments in a) Right-d), f)-i) and N=4 biological independent experiments in a) Left and e). Loading control: α-Tubulin in b) and i). Source data are provided as a Source data file.

Supplementary Figure 6

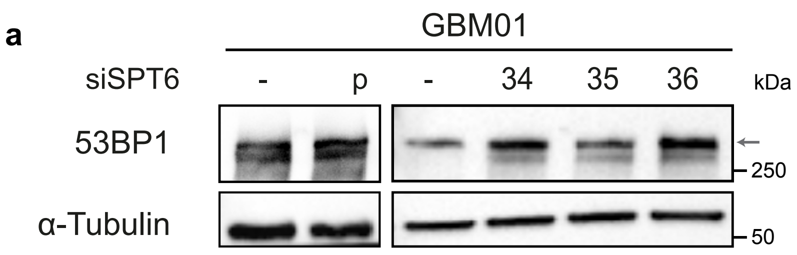

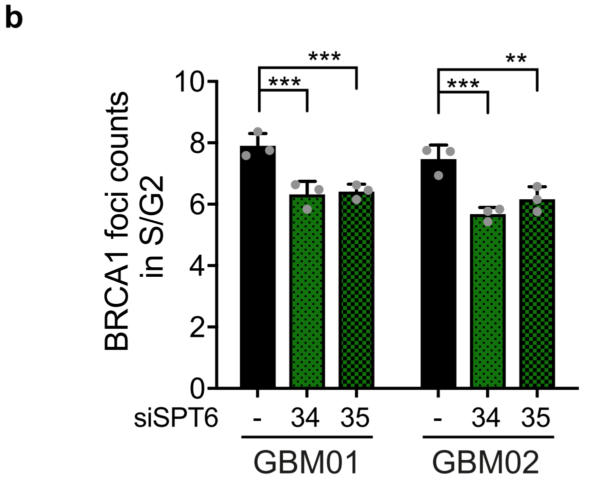

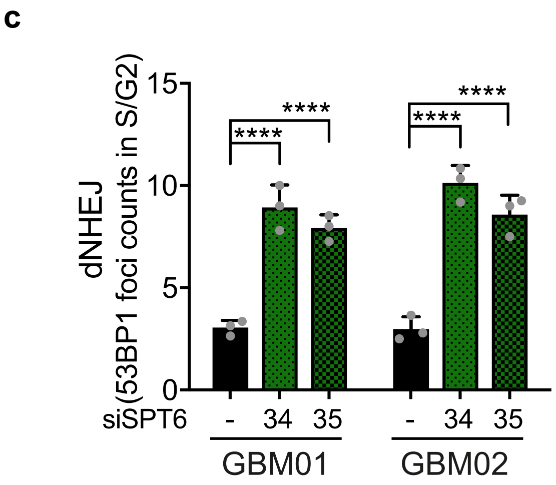

Supplementary Fig. 6: Figure providing supplementary information to main Figure 5e-m

(a) Representative immunoblot analysis of 53BP1 protein expression in GSCs (GBM01) transfected with siCON, siSPT6-p or 3 independent siRNAs (siSPT6-34, siSPT6-35, siSPT6-36).

Loading control: α-Tubulin.

(**b**) BRCA1 foci quantification in S/G2 phase GSCs transfected with siCON or 2 independent siRNAs (siSPT6-34, siSPT6-35). Data are presented as mean ± s.d. GBM01: ****p*=0.0004 and ****p*=0.0007 and GBM02: ****p*=0.0001 and ***p*=0.0019; two-way ANOVA analysis followed by Dunnett's multiple comparison test.

(**c**) Quantification of dNHEJ in GSCs transfected with siCON or 2 independent siRNAs (siSPT6-34, siSPT6-35). Data are presented as mean ± s.d. *****p*<0.0001; two-way ANOVA analysis followed by Dunnett's multiple comparison test.

N=3 biological independent experiments in a)-c). Source data are provided as a Source data file.

Supplementary Figure 7

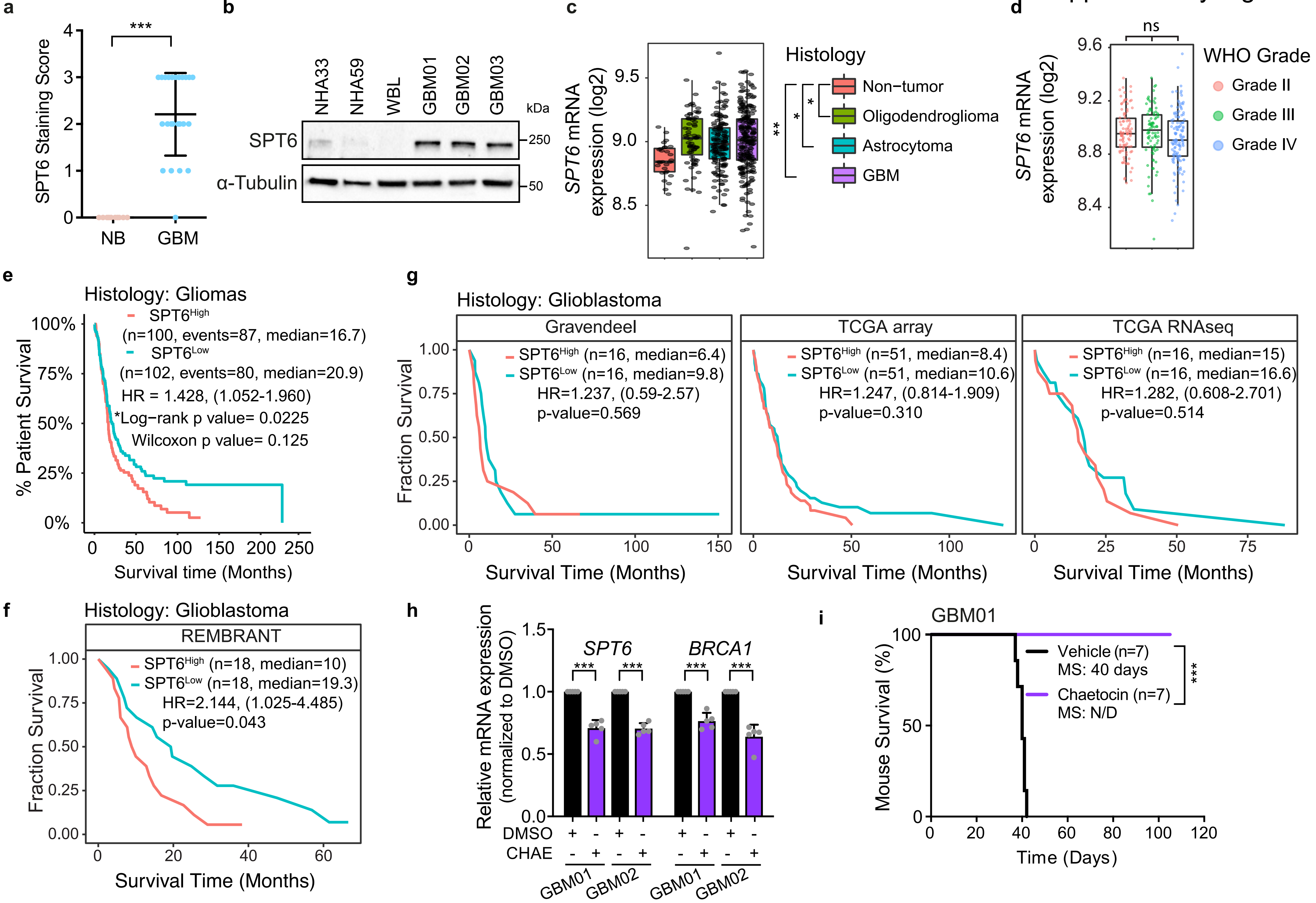

Supplementary Fig. 7: Figure providing supplementary information to main Figure 7

(**a**) Graph summarizing the results of IHC analysis of SPT6 expression in normal brain controls (NB, n=10) and GBM (n=24). ****p<0.001; two-tailed unpaired t-test.

(**b**) Representative immunoblot analysis of SPT6 expression in normal human astrocyte (NHA33, NHA59), whole brain lysate (WBL), GBM01, GBM02 and GBM03 xenograft lines. Loading control: α-Tubulin.

(**c**) *SPT6* expression analysis of REMBRANDT data (the National Cancer Institute's repository available via GlioVis) in non-tumor (n=28), oligodendroglioma (n=67), astrocytoma (n=147) and GBM (n=219). Center line of the box represent median; box bounds represent 25th and 75th percentiles and whiskers were calculated using Tukey's method. *p=0.012 (Oligodendroglioma), *p=0.0245 (Astrocytoma) and **p=0.0049 (GBM,); one-way ANOVA analysis followed by Tukey's multiple comparison test.

(**d**) *SPT6* expression analysis of REMBRANDT data in WHO grade II (n=54), III (n=47) and IV (n=71) gliomas. Center line of the box represent median; box bounds represent 25th and 75th percentiles and whiskers were calculated using Tukey's method. Non-significant (ns) p<0.05; one-way ANOVA analysis followed by Tukey's multiple comparison test.

(**e**) Analysis of REMBRANDT data indicates a positive correlation between $SPT6_{high}$ expression and poor survival in gliomas (Log–rank p-value=0.0225).

(**f**) Analysis of REMBRANDT data set indicates a positive correlation between $SPT6_{high}$ expression and poor survival in glioblastoma (Log–rank p-value=0.043).

(**g**) Analysis of Gravendeel, TCGA array and TCGA RNAseq data sets (the National Cancer Institute's repositories available via GlioVis) examining the correlation between $SPT6_{high}$ expression and glioblastoma patient survival.

(**h**) qRT-PCR analysis of *SPT6* and *BRCA1* mRNA expression in GSCs (GBM01, GBM02) treated with DMSO or chaetocin (CHAE). House-keeping control gene: HPRT. Data are normalized to DMSO control and presented as mean ± s.d. ****p<0.001; two-way ANOVA analysis followed by Sidak's multiple comparisons test.

(**i**) Kaplan-Meier survival curves for mice implanted with GBM01 GSCs treated cells with vehicle (DMSO) or 30 nM CHAE 24 hours prior implantation (n= 7 mice per group). MS= Median Survival and ND= Non-Determined. Statistical significance was tested using Log-rank/Mantel-Cox test. ***p=0.0004.

N=3 and N=5 biological independent experiments in b) and h), respectively. Source data are provided as a Source data file.

Supplementary Figure 8

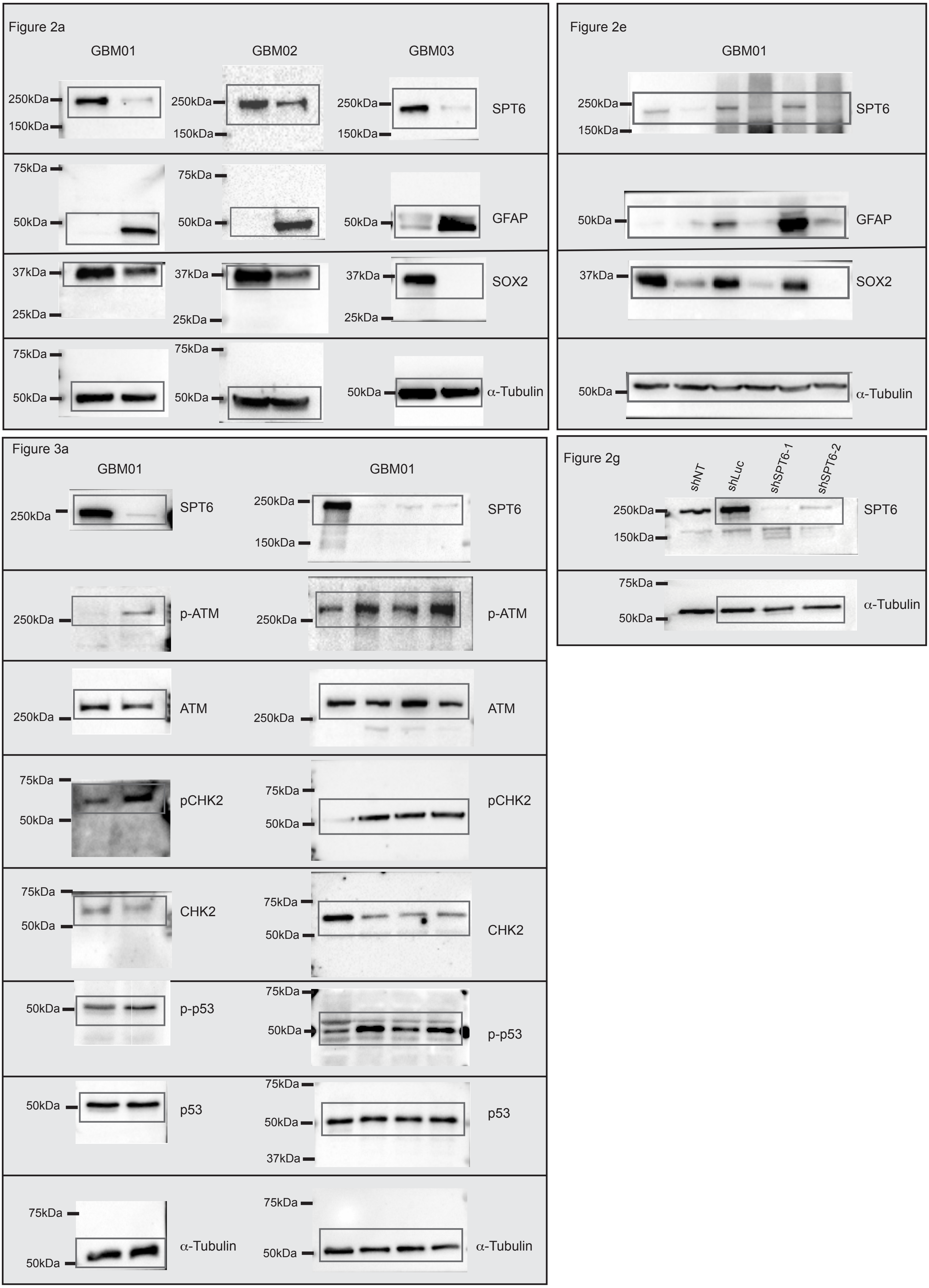

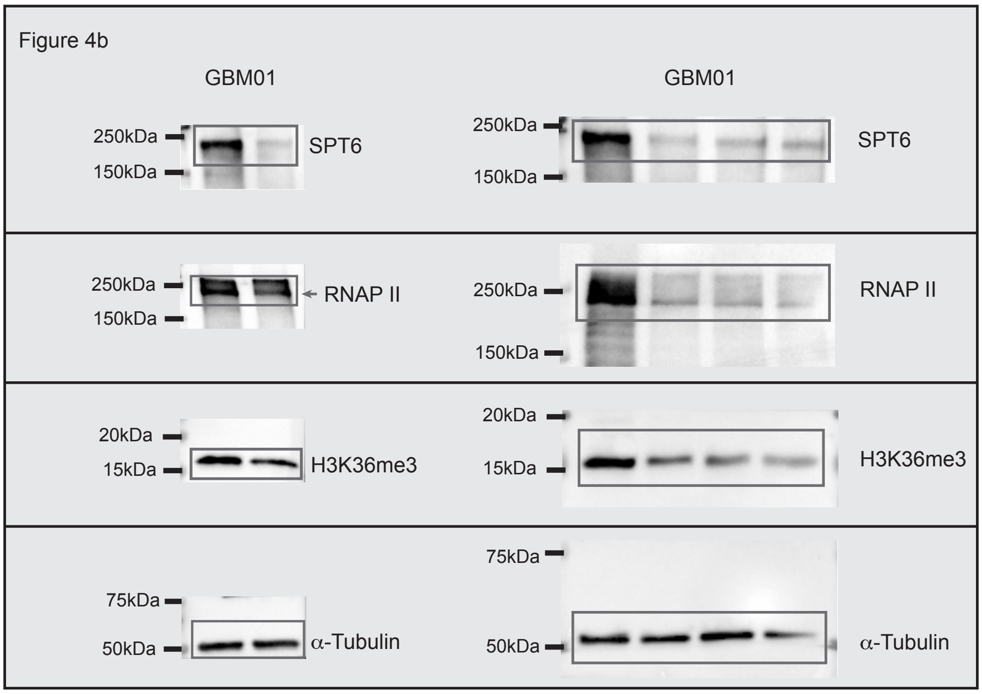
Figure 4b
GBM01
GBM01
250kDa
150kDa
SPT6
250kDa
150kDa
SPT6
250kDa
150kDa
RNAP II
250kDa
150kDa
RNAP II
20kDa
15kDa
H3K36me3
20kDa
15kDa
H3K36me3
75kDa
50kDa
α-Tubulin
75kDa
50kDa
α-Tubulin

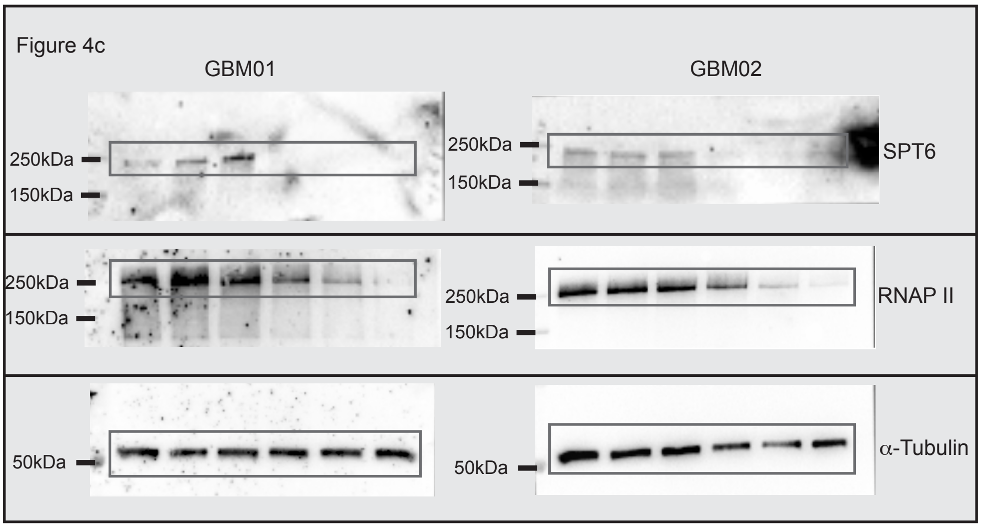
Figure 4c
GBM01
GBM02
250kDa
150kDa
250kDa
150kDa
SPT6
250kDa
150kDa
250kDa
150kDa
RNAP II
50kDa
50kDa
α-Tubulin

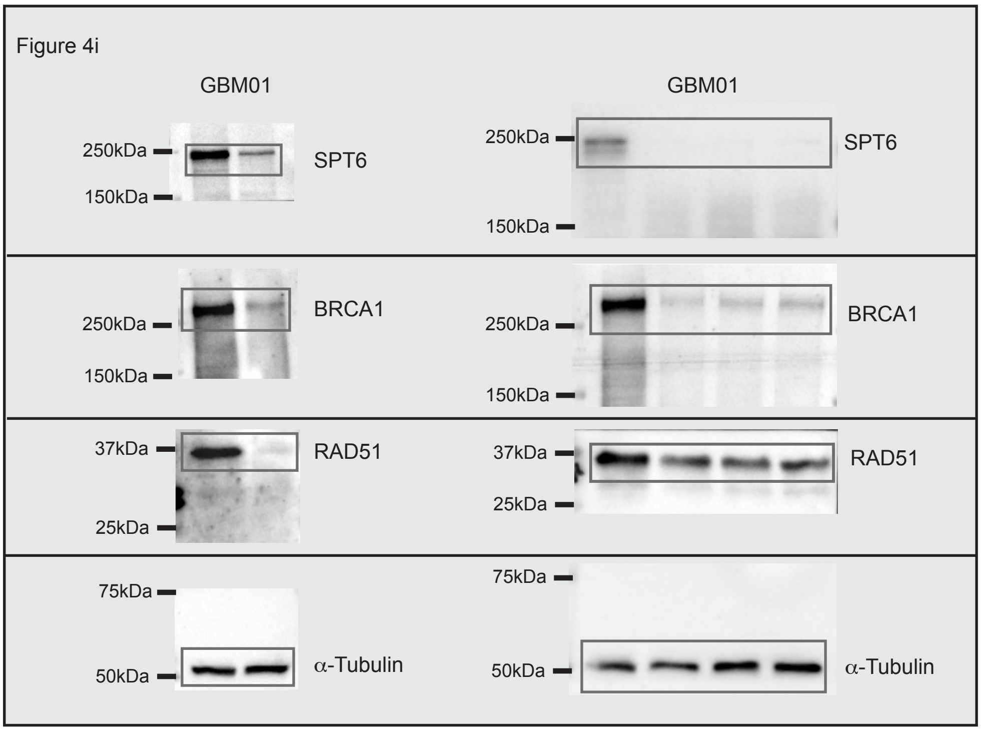
Figure 4i
GBM01
GBM01
250kDa
150kDa
SPT6
250kDa
150kDa
SPT6
250kDa
150kDa
BRCA1
250kDa
150kDa
BRCA1
37kDa
25kDa
RAD51
37kDa
25kDa
RAD51
75kDa
50kDa
α-Tubulin
75kDa
50kDa
α-Tubulin

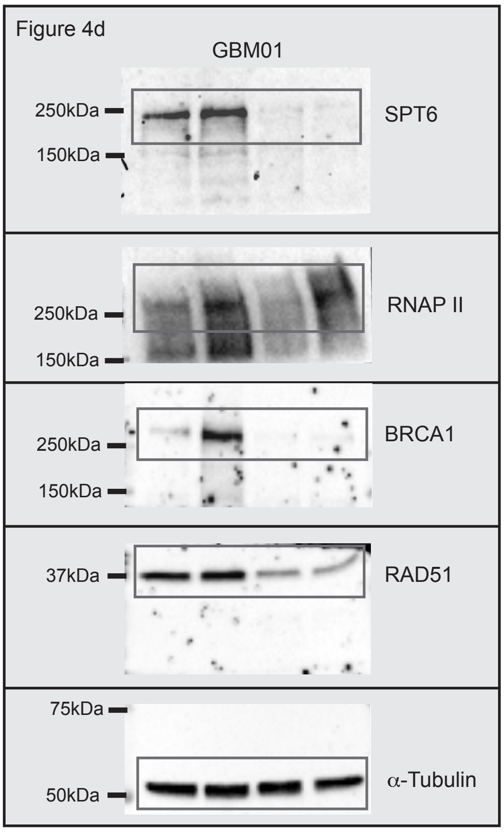
Figure 4d
GBM01
250kDa
150kDa
SPT6
250kDa
150kDa
RNAP II
250kDa
150kDa
BRCA1
37kDa
RAD51
75kDa
50kDa
α-Tubulin

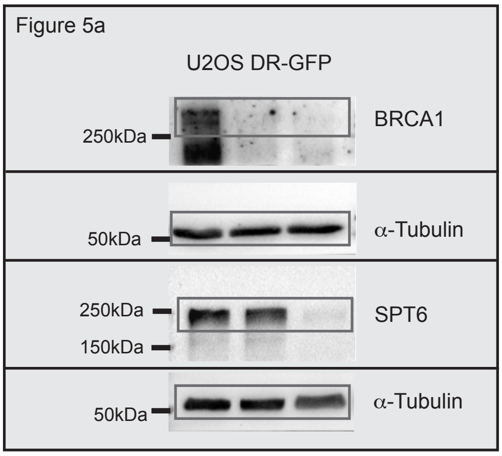
Figure 5a
U2OS DR-GFP
250kDa
BRCA1
50kDa
α-Tubulin
250kDa
150kDa
SPT6
50kDa
α-Tubulin

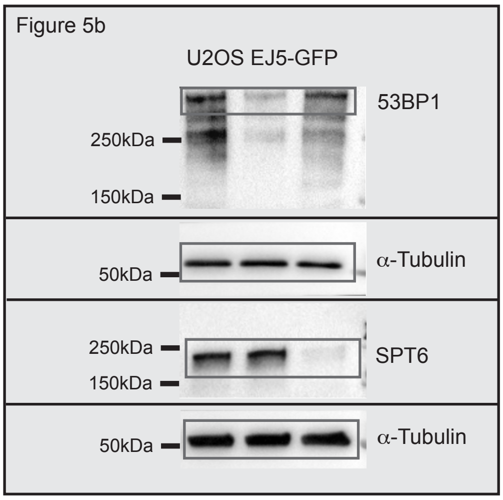
Figure 5b
U2OS EJ5-GFP
53BP1
250kDa
150kDa
50kDa
α-Tubulin
250kDa
150kDa
SPT6
50kDa
α-Tubulin

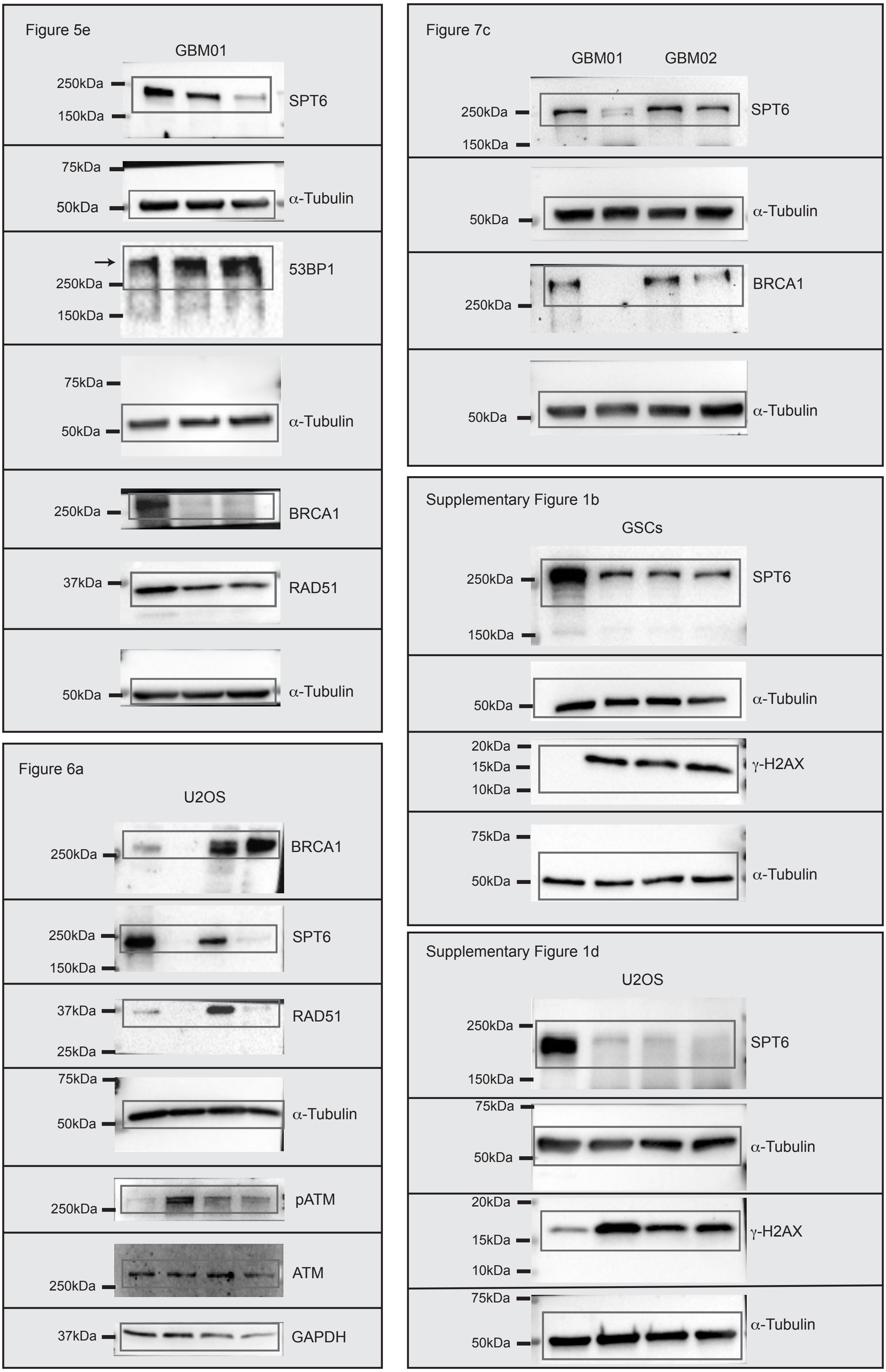
Figure 5e
GBM01
250kDa
150kDa
SPT6
75kDa
50kDa
α-Tubulin
53BP1
250kDa
150kDa
75kDa
50kDa
α-Tubulin
250kDa
BRCA1
37kDa
RAD51
50kDa
α-Tubulin
Figure 6a
U2OS
250kDa
BRCA1
250kDa
150kDa
SPT6
37kDa
25kDa
RAD51
75kDa
50kDa
α-Tubulin
250kDa
pATM
250kDa
ATM
37kDa
GAPDH
Figure 7c
GBM01
GBM02
250kDa
150kDa
SPT6
50kDa
α-Tubulin
250kDa
BRCA1
50kDa
α-Tubulin
Supplementary Figure 1b
GSCs
250kDa
150kDa
SPT6
50kDa
α-Tubulin
20kDa
15kDa
10kDa
γ-H2AX
75kDa
50kDa
α-Tubulin
Supplementary Figure 1d
U2OS
250kDa
150kDa
SPT6
75kDa
50kDa
α-Tubulin
20kDa
15kDa
10kDa
γ-H2AX
75kDa
50kDa
α-Tubulin

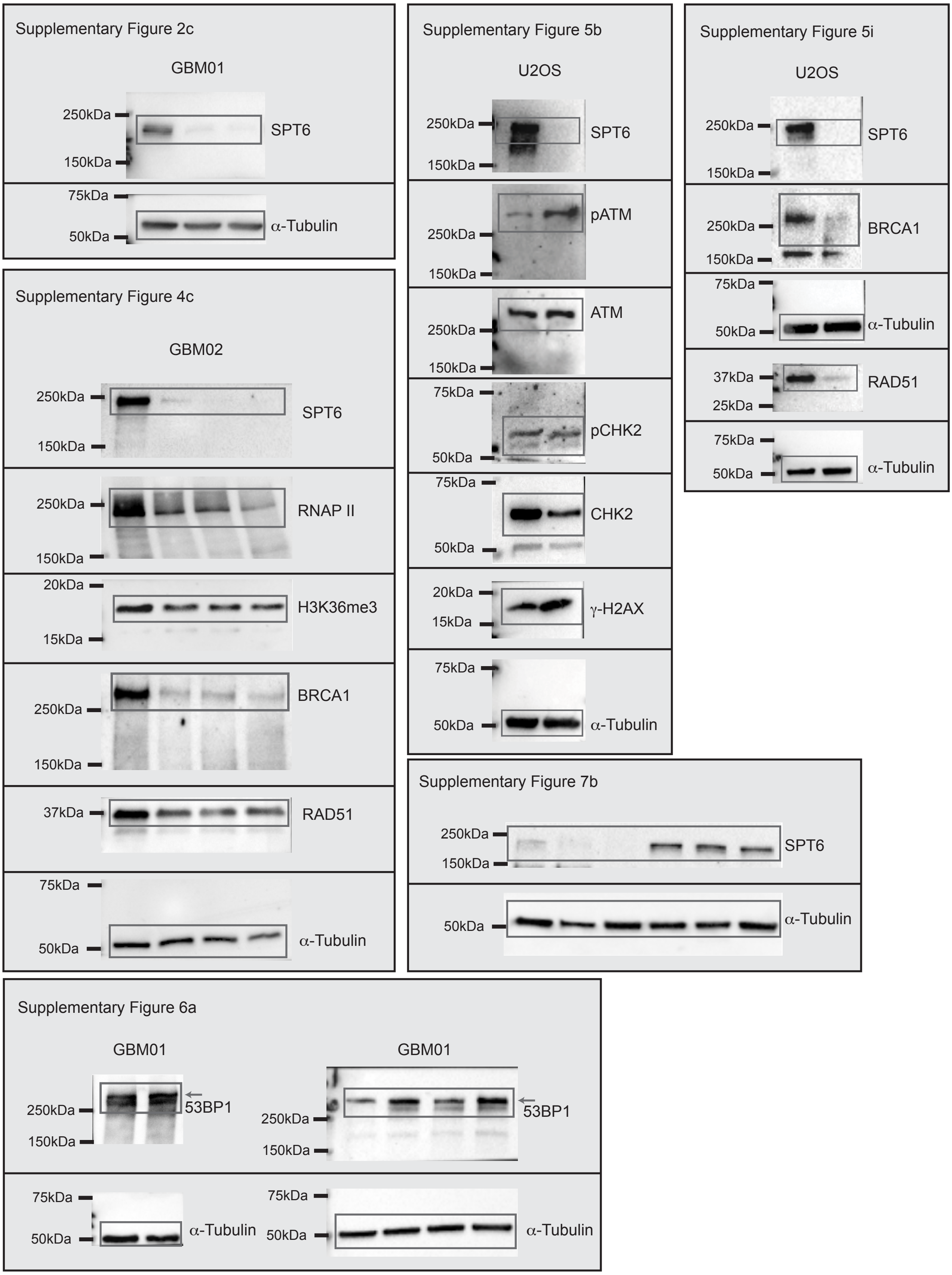

Supplementary Fig. 8: Uncropped images of immunoblots

Uncropped images of immunoblots are shown in the order presented in the Figures and Supplmentary Figures

Supplementary Figure 9

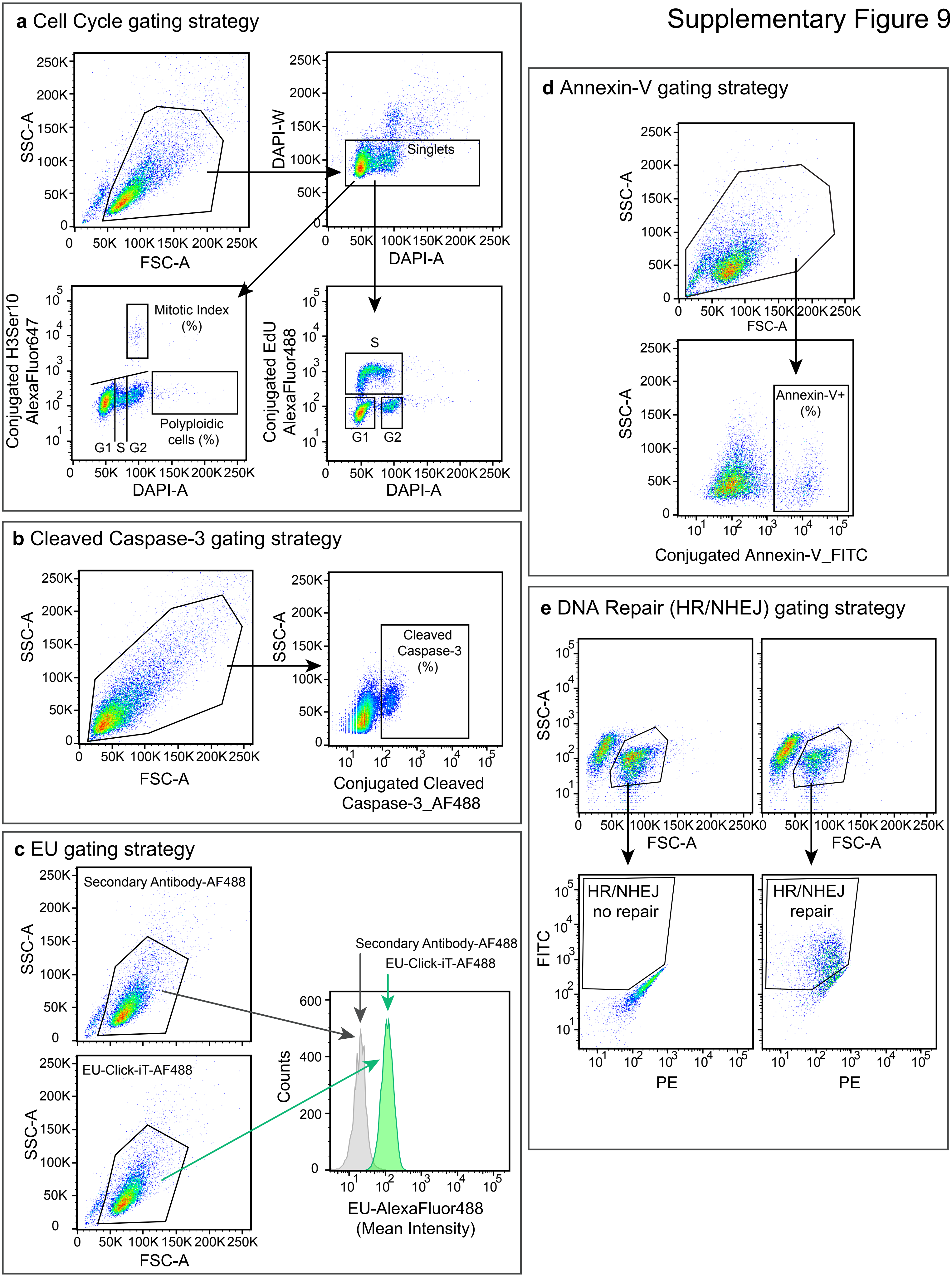

Supplementary Fig. 9: Gating Strategies for all FACS assays

(**a**) Cell Cycle gating strategy for Figures 3b-d, 5d, 5k and Supplementary Figures 3a-c, 5d.

(**b**) Cleaved Caspase-3 gating strategy for Figure 3f and Supplementary figure 5g

(**c**) EU gating strategy for Figure 4a and Supplementary Figure 4b

(**d**) Annexin-V gating strategy for Figure 3e and Supplementary Figure 5h

(**e**) DNA repair gating strategy for Figure 5h and 5i.

Supplementary Table 1 List of all antibodies used in this study

1_{ry} or 2_{ry}	Antibodies	Source	Catalog	Application
1_{ry}	SPT6	Abcam	Ab32820	WB, IF
1_{ry}	SPT6	Novus Biologicals	NB100-2582	ChIP
1_{ry}	ATM (D2E2)	Cell Signaling Technology	CTS-2873S	WB
1_{ry}	pATM (phospho-Ser1981)	GeneTex	GTX61739	WB
1_{ry}	CHK2 (DCS-273)	Santa Cruz Biotechnology	sc-56297	WB
1_{ry}	pCHK2 (phospho-Thr68)	Cell Signaling Technology	CTS-2661	WB
1_{ry}	p53 (DO-1)	Santa Cruz Biotechnology	sc-126	WB
1_{ry}	p-p53 (Phospho-Ser15)	Cell Signaling Technology	CTS-9284	WB
1_{ry}	Histone H2A.X (phospho-Ser139)	Millipore	05-636	WB, IF
1_{ry}	53BP1	Abcam	ab36823	WB, IF
1_{ry}	RNAPII (8WG16)	Covance Research Products	MMS-126R	WB
1_{ry}	H3K36me3	Abcam	ab9050	WB
1_{ry}	SOX-2	R&D Systems	AF2018	WB
1_{ry}	GFAP	Agilent	Z0334	WB
1_{ry}	BRCA1 (D-9)	Santa Cruz Biotechnology	sc-6954	WB, IF
1_{ry}	RAD51	Abcam	ab213	WB
1_{ry}	RAD51	Abcam	ab6380	WB
1_{ry}	cyclin A (H-432)	Santa Cruz Biotechnology	sc-751	IF
1_{ry}	alpha-Tubulin	Sigma-Aldrich	T9026	WB
1_{ry}	Cleaved Caspase-3 (Asp175) (Alexa Fluor$_{(R)}$ 488 Conjugate)	Cell Signaling Technology	CTS-9669	FACS
1_{ry}	Histone H3, phospho (Ser10) (Alexa Fluor$_{(R)}$ 647 Conjugate)	Cell Signaling Technology	CTS-9716	FACS
1_{ry}	IgG, purified (serum non-immune)	Alpha Diagnostic International	20009-5	ChIP
2_{ry}	Horse Anti-Mouse IgG (H+L), peroxidase	Vector Laboratories	PI-2000	WB
2_{ry}	Goat Anti-Rabbit IgG (H+L), peroxidase	Vector Laboratories	PI-1000	WB
2_{ry}	Goat Anti-Rabbit IgG (H+L), peroxidase	Vector Laboratories	PI-9500	WB
2_{ry}	Goat Anti-Mouse IgG (H+L) Highly Cross-adsorbed, Alexa Fluor 568	Molecular Probes	A-11031	IF
2_{ry}	Goat anti-Rabbit IgG (H+L) Highly Cross-adsorbed, Alexa Fluor 488	Molecular Probes	A-11034	IF
2_{ry}	Goat Anti-Rabbit IgG (H+L) Highly Cross-adsorbed, Alexa Fluor 568	Molecular Probes	A-11036	IF
2_{ry}	Goat Anti-Mouse IgG (H+L) Highly Cross-adsorbed, Alexa Fluor 488	Molecular Probes	A-11029	IF

Supplementary Table 2 **List of qPCR Primers used in this study**

Target gene	Primer Sequence-sense (5'->3')	Primer Sequence-antisense (5'->3')
SPT6	TCACCACCCCTCAGTACCAC	CTGCATGGCTGTTGGACTT
BRCA1	ACTGCAGCCAGCCACAGGTA	TAGCCAGGACAGTAGAAGGA
RAD51	TGAGGGTACCTTTAGGCCAGA	CACTGCCAGAGAGACCATACC
HPRT1	TGGCCATCTGCTTAGTAGAG	AACAACAATCCGCCCAAAGG
BRCA1 promoter	TCTTAGTGTGACGTGACCCC	AGGGACAAGTGGTAAGAGCC
