# Supplementary material for: SPT6-driven error-free DNA repair safeguards genomic stability of glioblastoma cancer stem-like cells
Source: Nat Commun. 2020 Sep 18;11:4709. doi: 10.1038/s41467-020-18549-8 (PMC7501306; doi:10.1038/s41467-020-18549-8)
Supplement: Supplementary file 2 — Description of Additional Supplementary Files [file 41467_2020_18549_MOESM2_ESM.pdf]

Description of Additional Supplementary Files

Title: Supplementary Data 1

Description: Chromatin remodeling siRNA library gene list

Title: Supplementary Data 2

Description: A list of genes scoring as 'hits' in GSCs and DGCs

Title: Supplementary Data 3

Description: Differential gene expression in SPT6 silenced GSCs

Title: Supplementary Data 4

Description: Gene set enrichment analysis in SPT6 silenced GSCs

Title: Supplementary Data 5

Description: Differential gene expression analysis of Suva et al (GSCs vs DGCs)

Title: Supplementary Data 6

Description: List of gene set clusters corresponding to a cluster bubble plot shown in Figure 4f

Supplementary Data 7

Description: List of candidates derived from CMap analysis shown in Figure 7b
