# Supplementary material for: SPT6-driven error-free DNA repair safeguards genomic stability of glioblastoma cancer stem-like cells
Source: Nat Commun. 2020 Sep 18;11:4709. doi: 10.1038/s41467-020-18549-8 (PMC7501306; doi:10.1038/s41467-020-18549-8)
Supplement: Supplementary file 10 — Reporting Summary [file 41467_2020_18549_MOESM10_ESM.pdf]

nature research

Corresponding author(s): Hamerlik Petra

Last updated by author(s): Aug 27, 2020

Reporting Summary

Nature Research wishes to improve the reproducibility of the work that we publish. This form provides structure for consistency and transparency in reporting. For further information on Nature Research policies, see Authors & Referees and the Editorial Policy Checklist .

Statistics

For all statistical analyses, confirm that the following items are present in the figure legend, table legend, main text, or Methods section.

n/a	Confirmed	
☐	☒	The exact sample size (*n*) for each experimental group/condition, given as a discrete number and unit of measurement
☐	☒	A statement on whether measurements were taken from distinct samples or whether the same sample was measured repeatedly
☐	☒	The statistical test(s) used AND whether they are one- or two-sided<br>*Only common tests should be described solely by name; describe more complex techniques in the Methods section.*
☒	☐	A description of all covariates tested
☐	☒	A description of any assumptions or corrections, such as tests of normality and adjustment for multiple comparisons
☐	☒	A full description of the statistical parameters including central tendency (e.g. means) or other basic estimates (e.g. regression coefficient) AND variation (e.g. standard deviation) or associated estimates of uncertainty (e.g. confidence intervals)
☐	☒	For null hypothesis testing, the test statistic (e.g. *F*, *t*, *r*) with confidence intervals, effect sizes, degrees of freedom and *P* value noted<br>*Give P values as exact values whenever suitable.*
☒	☐	For Bayesian analysis, information on the choice of priors and Markov chain Monte Carlo settings
☒	☐	For hierarchical and complex designs, identification of the appropriate level for tests and full reporting of outcomes
☐	☒	Estimates of effect sizes (e.g. Cohen's *d*, Pearson's *r*), indicating how they were calculated

Our web collection on statistics for biologists contains articles on many of the points above.

Software and code

Policy information about availability of computer code

Data collection	ScanR acquisition software for Olympus high-content screening microscope: used for image acquisition in miscroscopy-based foci quantification experiments<br>Zeiss ZEN software, for images taken in Zeiss LSM800 confocal microscope: used for the acquisition of SPT6 co-location with H2AXSer139 staining experiment<br>BD FACSuite V1.0.6 software: used for the acquisition of all flow cytometry data<br>Comet Assay IV software: used for the comet assay experiments (microphotographs and tail moments)<br>BioRad Image Lab 5.2.1: used for image acquisition of protein immunoblots
Data analysis	ScanR analysis software from Olympus: used for image analysis in miscroscopy-based foci quantification experiments<br>FlowJo _ version 10.6.0: used for the analysis of all flow cytometry data<br>Comet Assay IV software: used for the analysis of comet assay data<br>Extreme Limiting Dilution Analysis (ELDA) software (http://bioinf.wehi.edu.au/software/elda): analysis of cancer stem-like frequency<br>Microsoft Excel 2016 and GraphPad Prism 8: used for numerical and statistical analysis<br>GlioVis data portal (http://gliovis.bioinfo.cnio.es/): used for survival analysis using publicly available datasets- http://<br>gliovis.bioinfo.cnio.es/

For manuscripts utilizing custom algorithms or software that are central to the research but not yet described in published literature, software must be made available to editors/reviewers. We strongly encourage code deposition in a community repository (e.g. GitHub). See the Nature Research guidelines for submitting code & software for further information.

Data

Policy information about availability of data

All manuscripts must include a data availability statement. This statement should provide the following information, where applicable:

- Accession codes, unique identifiers, or web links for publicly available datasets
- A list of figures that have associated raw data
- A description of any restrictions on data availability

The raw FASTQ files, as well as the gene count matrix have been submitted to NCBI's Gene Expression Omnibus (GEO) and have been assigned the accession number GSE125621 and permanent URL: https://www.ncbi.nlm.nih.gov/geo/query/acc.cgi?acc=GSE125621.
The TCGA array, TCGA RNAseq, Rembrant and Gravendeel database can be downloaded from GlioVis data portal (http://gliovis.bioinfo.cnio.es/). All other data supporting the findings of this study are available within the article and its supplementary information and data files. The source data underlying Figures. 1a, 1d,2b-d, 2f, 3b-h, 4a, 4h, 4j, 5a-d, 5f-m, 6b-d, 7d-g and Supplementary Figures 1a, 1c, 2a-b, 2d, 3a-f, 4b, 4e, 4g, 5a, 5c-h, 6b-c, 7a,7c-d and 7h-i, are provided as a Source Data file.

Field-specific reporting

Please select the one below that is the best fit for your research. If you are not sure, read the appropriate sections before making your selection.

☒ Life sciences ☐ Behavioural & social sciences ☐ Ecological, evolutionary & environmental sciences

For a reference copy of the document with all sections, see nature.com/documents/nr-reporting-summary-flat.pdf

Life sciences study design

All studies must disclose on these points even when the disclosure is negative.

Sample size	Sample size in in vitro experiments were chosen based on previous experience with similar experiments, or in the case of retrospective analysis , all available samples were included.<br>For human retrospective IHC study: we have used already available human GBM cohort, where the patients were identified through the database of the University Hospital Olomouc, CZ.<br>For animal studies: To estimate the minimal number of animals per arm necessary to achieve statistical significance, we use the following formula : N=1+2C(s/d)^2 where n=number of animals per arm, C=7.85 when alpha=0.05 and 1-beta=0.8 (significance level of 5% with a power of 80%), s= standard deviation, and d=difference to be detected. The precise number of animals used are described in each figure legend. Each study was designed to minimize unnecessary animal use, optimize statistical power, and account for known variance in each model system. The precise number of animals used are described in each figure legend.<br>In in vitro experiments, the sample size was chosen based on previous experience with similar experiments, and in order to reproducibly detect specific effects. No statistical method was used to predetermine sample size for the in vitro experiments, but all in vitro experiments were repeated three times.
Data exclusions	No data were excluded in vitro or in vivo studies. To improve the visual presentation 2 "unclear" unconnected clusters as well as the following 3 unconnected clusters were removed from the visualization: Aminoacytelation, Monosaccharide processes and ERBB2 and MEK signaling. The entire process is documented in Supplementary Data 6.
Replication	The reproducibility for each analysis was confirmed by three independent experiments.
Randomization	All samples such as cells or animals were randomly allocated into experimental groups.
Blinding	All animal experiments were fully blinded. Animal caretakers responsible for the drug administration and the termination of animals at the onset of neurological deficits were not given the information which arm was vehicle control and which drug chaetocin.<br>No blinding for in vitro experiments in this study. All analyses were performed using quantifiable parameters (e.g. luciferase signal values for cell viability assay analysis, tumorsphere number value for tumorsphere formation assay, etc), so no bias was possible.

Reporting for specific materials, systems and methods

We require information from authors about some types of materials, experimental systems and methods used in many studies. Here, indicate whether each material, system or method listed is relevant to your study. If you are not sure if a list item applies to your research, read the appropriate section before selecting a response.

Materials & experimental systems

n/a	Involved in the study	
	☒	Antibodies
	☒	Eukaryotic cell lines
☒		Palaeontology
	☒	Animals and other organisms
	☒	Human research participants
☒		Clinical data

Methods

n/a	Involved in the study	
☒		ChIP-seq
	☒	Flow cytometry
☒		MRI-based neuroimaging

Antibodies

Antibodies used

Primary antibodies
SPT6 (Rabbit polyclonal, Abcam, Cat#ab32820)
SPT6 (Rabbit polyclonal, Novus Biologicals, Cat#NB100-2582)
ATM (Rabbit monoclonal, Cell Signaling Technology, Cat#2873S, Clone D2E2)
p-ATM (Ser-1981) (Rabbit monoclonal, GeneTex, Cat#61739, Clone EP1890Y)
CHK2 (Mouse monoclonal, Santa Cruz Biotechnology, Cat#sc-56297, Clone DCS-273)
pCHK2 (Thr68) (Rabbit polyclonal, Cell Signaling Technology, Cat#2661)
p53 (Mouse monoclonal, Santa Cruz Biotechnology, Cat#sc-126, Clone DO-1)
p-p53 (Ser15) (Rabbit polyclonal, Cell Signaling Technology, Cat#9284)
Histone H2A.X (Ser139) (Mouse monoclonal, Millipore, Cat#05-636, Clone JBW301)
53BP1 (Rabbit polyclonal, Abcam, Cat#ab36823)
RNAP II (Mouse monoclonal, Covance Research Products Inc, Cat#MMS-126R, Clone 8WG16)
H3K36me3 (Rabbit polyclonal, Abcam, Cat#ab9050)
Sox2 (Goat polyclonal, R&D Systems, Cat#AF2018)
GFAP (Rabbit polyclonal, Agilent, Cat#Z0334)
BRCA1 (Mouse monoclonal, Santa Cruz Biotechnology, Cat#sc-6954, Clone D-9)
RAD51 (Mouse monoclonal, Abcam, Cat# ab213, Clone 14B4)
RAD51 (Rabbit polyclonal, Abcam, Cat# ab3801)
cyclin A (Rabbit polyclonal, Santa Cruz Biotechnology, Cat# sc-751 H432)
α-Tubulin (Mouse monoclonal, Sigma-Aldrich, Cat#T9026, Clone DM1A)
Cleaved Caspase-3 (Asp175) (Alexa Fluor(R) 488 Conjugate, Rabbit polyclonal, Cell Signaling Technology, Cat# 9669)
p-H3 (Ser10) (Alexa Fluor(R) 647 Conjugate, Rabbit polyclonal, Cell Signaling Technology, Cat# 9716)
Rabbit IgG, purified (serum non-immune) (Alpha Diagnostic International, Cat#20009-5)

Secondary antibodies
Horse Anti-Mouse IgG (H+L), peroxidase (Vector Laboratories, Cat# PI-2000)
Goat Anti-Rabbit IgG (H+L), peroxidase (Vector Laboratories, Cat# PI-1000)
Goat Anti-Rabbit IgG (H+L), peroxidase (Vector Laboratories, Cat# PI-9500)
Goat Anti-Mouse IgG (H+L) Highly Cross-adsorbed Secondary Antibody, Alexa Fluor 488 (Molecular Probes, Cat# A-11029)
Goat Anti-Mouse IgG (H+L) Highly Cross-adsorbed Secondary Antibody Alexa Fluor 568 (Molecular Probes, Cat# A-11031)
Goat anti-Rabbit IgG (H+L) Highly Cross-adsorbed Secondary Antibody, Alexa Fluor 488 (Molecular Probes, Cat# A-11034)
Goat Anti-Rabbit IgG (H+L) Highly Cross-adsorbed Secondary Antibody, Alexa Fluor 568 (Molecular Probes, Cat# A-11036)

Validation

All primary antibodies used in this study were validated by manufacturers and/or frequently used in publications. Validation statement for each antibody is provided on the manufacture's website. All secondary antibodies are used in many publications. The detailed information is listed as follows:

SPT6 (Rabbit polyclonal, Abcam, Cat#ab32820). Abcam website antibody validation. Western Blot analysis of extracts from Hela and and Jurkat whole cell lysates, detecting 1 or 2 bands of approximately 230 kDa (predicted molecular weight 199 kDa). Citation from manufacturer are listed at https://www.abcam.com/spt6-antibody-ab32820.html

SPT6 (Rabbit polyclonal, Novus Biologicals, Cat#NB100-2582). ChIP reactivity reported in scientific literature (PMID:23503590). The immunogen recognized by this antibody maps to a region between residue 425 and 475 of human Suppressor of Ty 6 Homolog using the numbering given in entry NP_003161.2 (GeneID 6830). Citation from manufacturer are listed at https://www.novusbio.com/products/spt6-antibody_nb100-2582

ATM (Rabbit monoclonal, Cell Signaling Technology, Cat#2873S, Clone D2E2). CTS website antibody validation: Western Blot analysis of extracts from Hela, NCCIT and PYS2 cells. We cited this antibody in Rasmussen et al. 2016 PMID: 27845331. Citation from manufacturer are listed at https://www.cellsignal.com/products/primary-antibodies/atm-d2e2-rabbit-mab/2873.

p-ATM (Ser-1981) (Rabbit monoclonal, GeneTex, Cat#61739, Clone EP1890Y). We cited this antibody in Rasmussen et al. 2016 PMID: 27845331). Citation from "CiteAb" website are listed at https://www.citeab.com/antibodies/562913-gtx61739-atm-

phospho-ser1981-antibody-ep1890y

CHK2 (Mouse monoclonal, Santa Cruz Biotechnology, Cat#sc-56297, Clone DCS-273). Santa Cruz Biotechnology website antibody validation: Western Blot analysis of extracts from Hela, CCRF-CEM, K-562 and Jurkat whole cell lysates. We cited this antibody in Rasmussen et al. 2016 PMID: 27845331). Citation from manufacturer are listed at https://www.scbt.com/p/chk2-antibody-dcs-273

pCHK2 (Thr68) (Rabbit polyclonal, Cell Signaling Technology, Cat#2661). CTS website antibody validation: Western Blot analysis of extracts from Cos cells, untreated or UV-treated (100mJ/cm2, 1hour recovery). We cited this antibody in Rasmussen et al. 2016 PMID: 27845331). Citation from manufacturer are listed at https://www.cellsignal.com/products/primary-antibodies/phospho-chk2-thr68-antibody/2661

p53 (Mouse monoclonal, Santa Cruz Biotechnology, Cat#sc-126, Clone DO-1). Santa Cruz Biotechnology website antibody validation: Western Blot analysis of extracts from SW480, A549 and HUV-EC-C whole cell lysates. Citation from manufacturer are listed at https://www.scbt.com/es/p/p53-antibody-do-1

p-p53 (Ser15) (Rabbit polyclonal, Cell Signaling Technology, Cat#9284). CTS website antibody validation: Western Blot analysis of extracts from Mvllu cells treated with UV or hydroxyurea (20 mM) for the indicated times. Citation from manufacturer are listed at https://www.cellsignal.com/products/primary-antibodies/phospho-p53-ser15-antibody/9284

Histone H2A.X (Ser139) (Mouse monoclonal, Millipore, Cat#05-636, Clone JBW301). Merck/Millipore website antibody validation: 1) Western Blot analysis of extracts from Jurkat cells untreated or treated with staurospoine. 2) Immunofluorescence from Jurkat cells treated with etoposide. We cited this antibody in Rasmussen et al. 2016 PMID: 27845331). Citation from manufacturer are listed at https://www.merckmillipore.com/DK/en/product/Anti-phospho-Histone-H2A.X-Ser139-Antibody-clone-JBW301,MM_NF-05-636?ReferrerURL=https%3A%2F%2Fwww.google.com%2F

53BP1 (Rabbit polyclonal, Abcam, Cat#ab36823). Abcam website antibody validation: 1) Western Blot analysis of extracts from U2OS and 293T cells. 2) Immunofluorescence from normal MEFs and exposed to 10Gy of IR. Citation from manufacturer are listed at https://www.abcam.com/53bp1-antibody-ab36823.html

RNAP II (Mouse monoclonal, BioLegend, previously Covance Research Products Inc, Cat#MMS-126R, Clone 8WG16). BioLegend website antibody validation: Western Blot analysis of extracts from Hela, Jurkat and Raw cells. Citation from manufacturer are listed at https://www.biolegend.com/en-us/products/purified-anti-rna-polymerase-ii-antibody-11544

H3K36me3 (Rabbit polyclonal, Abcam, Cat#ab9050). Abcam website antibody validation: Western Blot analysis of extracts from L4440 control RNAi and sig-7 (RNAi) embryos. Citation from manufacturer are listed at https://www.abcam.com/histone-h3-tri-methyl-k36-antibody-chip-grade-ab9050.html#description_images_2

Sox2 (Goat polyclonal, R&D Systems, Cat#AF2018). R&D Systems website antibody validation: Western Blot analysis of lysates from D3 mouse embryonic stem cell line, NTera-2 human testicular embryonic carcinoma cell line, F9 mouse teratocarcinoma stem cells, and rat cortical stem cells. We cited this antibody in Wang et al 2018 PMID: 29625067. Citation from manufacturer are listed at https://www.rndsystems.com/products/human-mouse-rat-sox2-antibody_af2018

GFAP (Rabbit polyclonal, Agilent Dako, Cat#Z0334). We cited this antibody in Staberg et al 2018 (PMID: 29360266): Western blot analysis of extracts from GBM primary cells lines. Citation from manufacturer are listed at https://www.agilent.com/en/product/immunohistochemistry/antibodies-controls/primary-antibodies/glial-fibrillary-acidic-protein-(concentrate)-76683#productdetails

BRCA1 (Mouse monoclonal, Santa Cruz Biotechnology, Cat#sc-6954, Clone D-9). Santa Cruz Biotechnology website antibody validation: 1) Western Blot analysis of nuclear extracts from A-431, Hela and MCF7 cells. 2) Immunofluorescence from UVA laser-microirradiated Hela cells. Citation from manufacturer are listed at https://www.scbt.com/es/p/brca1-antibody-d-9

RAD51 (Mouse monoclonal, Abcam, Cat# ab213, Clone 14B4). Abcam website antibody validation: Western Blot analysis of extracts from Jurkat, Raji and NCI-H929 whole cells lysates. Citation from manufacturer are listed at https://www.abcam.com/rad51-antibody-14b4-ab213.html

RAD51 (Rabbit polyclonal, Abcam, Cat# ab3801). Abcam website antibody validation: Western Blot analysis of extracts from Hela cells. Citation from manufacturer are listed at https://www.abcam.com/rad51-antibody-ab63801.html

Cyclin A (Rabbit polyclonal, Santa Cruz Biotechnology, Cat# sc-751 H432). This antibody is discontinued by the company but it was validated in immunofluorescence in several publications as Sridhara et al. 2016 PMID: 28076779. Citation from manufacturer are listed at https://www.scbt.com/es/p/cyclin-a-antibody-h-432

α-Tubulin (Mouse monoclonal, Sigma-Aldrich, Cat#T9026, Clone DM1A). Sigma-Aldrich website antibody validation: Western Blot analysis of extracts from Hela, Jurkat, NIH-3T3, PC-12, RAT2, CHO, MDBK and MDCK cells. We cited this antibody in Rasmussen et al. 2016 PMID: 27845331. Citation from manufacturer are listed at https://www.sigmaaldrich.com/catalog/product/sigma/t9026?lang=en&region=DK

Cleaved Caspase-3 (Asp175) (Alexa Fluor(R) 488 Conjugate, Rabbit polyclonal, Cell Signaling Technology, Cat# 9669). CTS website antibody validation: Flow cytometric analysis of Jurkat cells untreated or treated with etoposide. Citation from manufacturer are listed at https://www.cellsignal.com/products/antibody-conjugates/cleaved-caspase-3-asp175-antibody-alexa-fluor-488-

conjugate/9669

p-H3 (Ser10) (Alexa Fluor(R) 647 Conjugate, Rabbit polyclonal, Cell Signaling Technology, Cat# 9716). This antibody is discontinued by the company but it was validated in Flow cytometry in several publications as Hui-Ju Hsieh et al 2018 PMID: 30266942. We cited this antibody in Pedersen et al 2020 PMID: 32111042. Citation from manufacturer are listed at https://www.cellsignal.com/products/antibody-conjugates/phospho-histone-h3-ser10-antibody-alexa-fluor-647-conjugate/9716

Secondary Antibodies

Horse Anti-Mouse IgG (H+L), peroxidase (Vector Laboratories, Cat# PI-2000). Citation from manufacturer are listed at https://vectorlabs.com/peroxidase-horse-anti-mouse-igg-antibody.html

Goat Anti-Rabbit IgG (H+L), peroxidase (Vector Laboratories, Cat# PI-1000). Citation from manufacturer are listed at https://vectorlabs.com/peroxidase-goat-anti-rabbit-igg-antibody.html

Goat Anti-Rabbit IgG (H+L), peroxidase (Vector Laboratories, Cat# PI-9500). Citation from manufacturer are listed at https://vectorlabs.com/peroxidase-horse-anti-goat-igg-antibody.html

Goat Anti-Mouse IgG (H+L) Highly Cross-adsorbed Secondary Antibody, Alexa Fluor 488 (Molecular Probes, Cat# A-11029). Citation from manufacturer are listed at https://www.thermofisher.com/antibody/product/Goat-anti-Mouse-IgG-H-L-Highly-Cross-Adsorbed-Secondary-Antibody-Polyclonal/A-11029

Goat Anti-Mouse IgG (H+L) Highly Cross-adsorbed Secondary Antibody, Alexa Fluor 568 (Molecular Probes, Cat# A-11031). Citation from manufacturer are listed at https://www.thermofisher.com/antibody/product/Goat-anti-Mouse-IgG-H-L-Highly-Cross-Adsorbed-Secondary-Antibody-Polyclonal/A-11031

Goat anti-Rabbit IgG (H+L) Highly Cross-adsorbed Secondary Antibody, Alexa Fluor 488 (Molecular Probes, Cat# A-11034). Citation from manufacturer are listed at https://www.thermofisher.com/antibody/product/Goat-anti-Rabbit-IgG-H-L-Highly-Cross-Adsorbed-Secondary-Antibody-Polyclonal/A-11034

Goat Anti-Rabbit IgG (H+L) Highly Cross-adsorbed Secondary Antibody, Alexa Fluor 568 (Molecular Probes, Cat# A-11036). Citation from manufacturer are listed at https://www.thermofisher.com/antibody/product/Goat-anti-Rabbit-IgG-H-L-Highly-Cross-Adsorbed-Secondary-Antibody-Polyclonal/A-11036

Eukaryotic cell lines

Policy information about cell lines

Cell line source(s)	Primary GBM lines: GBM01, GBM02 and GBM03 were maintained as PDX lines derived from GBM tissue biopsies. GBM01 was a kind gift from Professor Jeremy Rich (Cleveland Clinic) and GBM02 and GBM03 were generated in our laboratory. Glioblastoma cancer-stem like cells (GSCs) and Differentiated GBM cells (DGCs) were isolated from these primary GBM lines by magnetic (MACS) sorting using CD133 microbeads kit.<br>Normal Human Astrocytes-Hippocampal cells (NHA33 and NHA59) were purchased from 3H biomedical (SC1830, 3H Biomedical) and maintained at low passages (max passage no.5)<br>Stable cell lines:<br>U2-OS cells were obtained from ATCC (HTB-96)<br>HEK-293T cells were cells were obtained from ATCC (CRL-11268)<br>U2-OS GFP cells were established in-house<br>U2-OS BRCA1-GFP cells were a gift from Prof. Jiri Bartek (Danish Cancer Society)<br>U2OS-EJ5-GFP and U2OS-DR-GFP cells were a gift from Dr. Pablo Huertas (University of Seville)
Authentication	All cell lines were authenticated using STR profiling.
Mycoplasma contamination	All cell lines used in this study were tested for mycoplasma contamination by PCR method. All cell lines used in this study were negative for mycoplasma.
Commonly misidentified lines<br>(See ICLAC register)	None of cell lines used in this study are listed by ICLAC.

Animals and other organisms

Policy information about studies involving animals; ARRIVE guidelines recommended for reporting animal research

Laboratory animals	Female NMRInu mice or female Balb/c nu/nu mice 6 weeks old at the time of intracranial tumor cell implantation; female NOG mice (Taconic,TAC;nog) for passaging of GBM lines as flank xenografts. Mice were housed under a 12 hours light/12 hours dark cycle in a temperature and humidity controlled and were fed ad libitum.
Wild animals	No wild animals were used.

Field-collected samples	No field-collected samples were used.
Ethics oversight	All animal studies were carried out according to Danish Welfare Law on Animal Experiments Act no 1306, protocol: 2012-15-2934-00636.

Note that full information on the approval of the study protocol must also be provided in the manuscript.

Human research participants

Policy information about studies involving human research participants

Population characteristics	Human glioblastoma (GBM) models derived from patient biopsies were histopathologically and clinically diagnosed.<br>Cells models maintained as PDX:<br>GBM01 were derived from a GBM from a male patient. GBM02 were derived from a male patient. GBM03 were derived from a male patient. The age range for these patients was 41-80.<br><br>Tissue sections used for IHC analysis: only diagnostic information was provided by the pathologist (WHO grade IV glioma, glioblastoma).
Recruitment	Only CNS tumor patients referred to the Dept. of Neurosurgery for surgical removal of CNS tumor were recruited at the time of their pre-surgery consultation by Associate Professor, Chief Physician Jane Skjøth-Rasmussen, MD, PhD at the Neurosurgical Department, Copenhagen University Hospital, Denmark. Human glioblastoma specimens were collected after the acquisition of a signed informed consent.
Ethics oversight	Protocol approved by the Regional Danish Ethical Committee/Danish Data Protection Agency (H-3-2009-136_63114).

Note that full information on the approval of the study protocol must also be provided in the manuscript.

Flow Cytometry

Plots

Confirm that:

- [x] The axis labels state the marker and fluorochrome used (e.g. CD4-FITC).
- [x] The axis scales are clearly visible. Include numbers along axes only for bottom left plot of group (a 'group' is an analysis of identical markers).
- [x] All plots are contour plots with outliers or pseudocolor plots.
- [x] A numerical value for number of cells or percentage (with statistics) is provided.

Methodology

Sample preparation	For cell cycle analysis, cells were pulse-labeled with EdU-AF488 conjugate for 30 min prior fixation and staining as outllined in Methods section. For cleaved-caspase 3 analysis, cells were fixed and stained as detailed in Methods section. For Annexin V analysis, cells were collected and stained without fixation as stated in Methods section. For the analysis of global transcription rates, cells were pulse-labeled with EU-probe for 1 hours prior to fixation and staining as detailed in Methods section.
Instrument	FACSVerse, BD Biosciences
Software	Flow data was analyzed using FlowJo software version 10.6.0
Cell population abundance	At least ten thousands events were recorded per sample on a FACSVerse flow cytometer.
Gating strategy	Each experimental population was gated to exclude cellular debris prior to analysis by plotting SSC-A vs FSC-A. The same gates were applied to both controls and experimental conditions. A representative figure of gating strategies for Cell Cycle, Cleaved Caspase-3, EU, Annexin-V and DNA repair assays is proved in Supplementary Information as Supplementary Figure 9

- [x] Tick this box to confirm that a figure exemplifying the gating strategy is provided in the Supplementary Information.
